# Amphidynamic Molecular Crystal with Temperature-Controlled Helical Hydrogen-Bonded Network: Proton Dynamics and Order–Disorder Phase Transition

**DOI:** 10.1021/acs.jpclett.6c00289

**Published:** 2026-03-24

**Authors:** Sylwia Zięba, Christelle Kadlec, Savita Priya, Yayi Lin, Adam Mizera, Martin Dressel, Petr Kužel

**Affiliations:** † Institute of Physics, Czech Academy of Sciences, Na Slovance 1999/2, 182 00 Prague 8, Czech Republic; ‡ Institute of Molecular Physics, 119441 Polish Academy of Sciences, M. Smoluchowskiego 17, 60-179 Poznan, Poland; § 1. Physikalisches Institut, 9149 Universität Stuttgart, Pfaffenwaldring 57, 70569 Stuttgart, Germany

## Abstract

Hydrated imidazolium hemimelitate with helical hydrogen bonding network is the first amphidynamic organic crystal observed in a group of imidazolium and carboxylic acid compounds. The sublattice of acid ions forms a static network, while the dynamic part comprises imidazole ions and water molecules. A transition from positional to orientational disorder of water molecules is observed as the temperature closes to room temperature and the spatial arrangement of cations leads to an order–disorder phase transition at a temperature of 150 K, which we analyzed in a wide spectral range using THz, FIR, MIR, and Raman spectroscopies. Furthermore, DFT calculations were employed to understand the molecular dynamics and the phase transition mechanism of the studied compound. The temperature-dependent spectra also revealed proton–phonon coupling to occur below 100 K. Our findings provide valuable information, such as temperature behavior of hydrogen bonds, anharmonicity, and coupling effects for the targeted design of amphidynamic materials.

## Graphical abstract

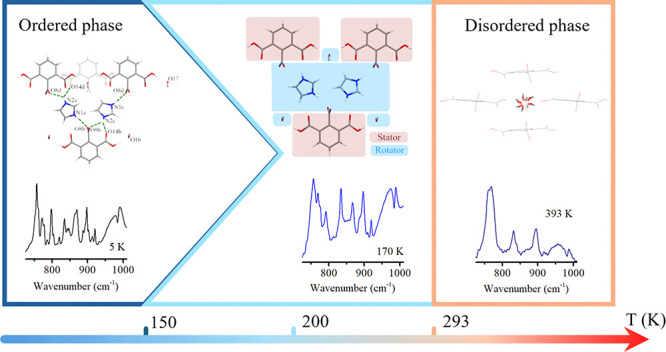

Amphidynamic organic materials (AOMs) form a group of organic crystals characterized by a coexistence of rigid and mobile structural elements (see Scheme S1).^1,2^ AOMs are then composed of static components with a larger moment of inertia (the stator) and rotating components with a smaller moment of inertia (the rotator).^3–5^ Rotators either have to possess a high rotational symmetry (as indicated in Scheme S1) or, in the case of small molecules like H_2_O, they should be allowed to alternate between commonly occupied sites in the structure.^6,7^ AOMs are therefore particularly attractive for designing and synthesizing novel molecular functional materials.^2–4,7^ The tunable and switchable relative motion of the components has potential applications in actuators, sensors, and shape memory materials.^1,4,8–11^

The rotator is activated by increasing the temperature, and a solid–solid (order–disorder) phase transition is observed simultaneously.^1^ Below the phase transition, the rotating component oscillates in the vicinity of the minimum of one of the potential wells. Above it, a sufficient thermal energy is available to overcome the energy barriers, resulting in a rotational motion as the molecule or its fragment jumps between equivalent orientations or equivalent lattice sites (i.e., the disorder mechanism).^12^ Dynamic molecules are characterized by long-range order with orientational or conformational disorder, which confers specific properties between solid state and plastic material to the high-temperature phase.^6,13,14^ The temperature of order–disorder phase transition depends on size and symmetry of the rotator, e.g, 360 K for NH_4_SCN,^13^ 200 K in TCNQ^0.5–^,^15^ or 156 K in [CN_3_NH_2_]­Zn­(HCOO)_3_ forming metal–organic frameworks.^16^

Hydrogen-bonding organic frameworks (HOFs) form one of the groups of materials with a potential to feature AOM properties due to their porous structure, which provides favorable conditions for a dynamic sublattice.^5,6,17^ HOFs are compounds with molecules connected by hydrogen bonds (HBs), which may frequently create a suitable structural environment to enable the molecular motion.^5,11,18^ The forces of crystal lattice cohesion, the possibility of proton transfer, and the amount of empty space available for the dynamic motion are all dependent on the energy of HBs.^11,14,19^ Therefore, they determine the crystal macroscopic properties, such as its electrical and thermal conductivity, and its thermal stability.^5,11,18,19^ With carboxylic acid salts and heterocyclic molecules, only two compounds are known to exhibit positional disorder (two positions of the imidazole ion are possible).^20–23^ However, no compound in which dynamic disorder occurs has been identified so far.

Intermolecular HBs are important for the orientational and conformational arrangement of molecules in organic and inorganic systems.^14,24^ They determine, to a large extent, the physical properties of the studied material upon temperature changes,^24,25^ namely the phase transition mechanisms^14^ and in many cases they dominate the overall dynamics of the system.^25^ The phase transitions are strongly influenced by molecular motion and weak intermolecular interactions.^11^ In the ordered phase, coupling effects such as phonon–phonon or proton–phonon couplings are important^1,18,19,24^ since they are considered as a significant anharmonic contribution to the phonon relaxation effects.^24^ A suitable method for analyzing these properties is molecular spectroscopy in a broad spectral range, such as a combination of THz, FIR, MIR, and Raman spectroscopies.^1,7,10,14^ ^,^ ^24–27^ Recently, it has been demonstrated that spectral analysis over a broad range enables the order–disorder transition mechanism to be explained.^19,25,27–29^ This approach elucidates the contribution of anharmonicity and various couplings to the behavior of HB networks in different phases and it appears particularly important in the study of compounds containing water molecules. The contribution of HBs to the physical properties of materials is still not completely clarified, which is particularly significant in the context of biological processes.^19,25,28^

This paper presents a temperature-dependent spectral analysis of hydrated imidazolium hemimelitate (HemImi·H_2_O) within the 10–3,500 cm^–1^ range by means of Terahertz (THz), infrared (MIR and FIR), and Raman spectroscopies. HemImi·H_2_O is a hydrated organic salt with a surprisingly high thermal stability up to 393 K and a maximum electrical conductivity of 10^–4^ S·m^–1^.^30^ Its high thermal stability is related to the helical network of N^+^–H···O^–^ and O–H···O^–^ HBs. For this reason our analysis of the thermal properties focuses on the bands linked to these HBs. Theoretical analysis of HBs was performed using the available crystallographic structure.^30^ The absorption and scattering spectra and their relation to the imidazole ion dynamics were interpreted within the framework of DFT calculations. We demonstrate that HemImi·H_2_O is the first amphidynamic crystal in the group of carboxylic acid salts with imidazole.

## Assessment of Modes at Room Temperature

HemImi·H_2_O is a salt composed of water molecules and imidazolium and acid ions in a 1:1:1 ratio, which crystallizes in a monoclinic system (Figure S1).^30^ The crystal structure has been described in *ref*.^30^ Water molecules and anions are connected by O–H···O^–^ and O–H···O HBs (Figures 1a and S1b) and cations and anions by N^+^–H···O^–^ HBs (Figures 1b and S1a). An ensemble of measured spectra by THz, FTIR and Raman spectroscopies at room temperature is shown in Figure 1c,d. Bands characteristic of the HBs within the spectral range of 10–3500 cm^–1^ are marked in the plots. Three characteristic ranges can be distinguished: 2900–3500 cm^–1^ associated with core vibrations (stretching of the N–H and O–H bonds); 500–1800 cm^–1^ associated with the ’fingerprint’ (deformational and stretching vibrations of bonds such as CO, COO); and 0–400 cm^–1^ associated with collective lattice vibrations (phonons).

**Figure 1:**
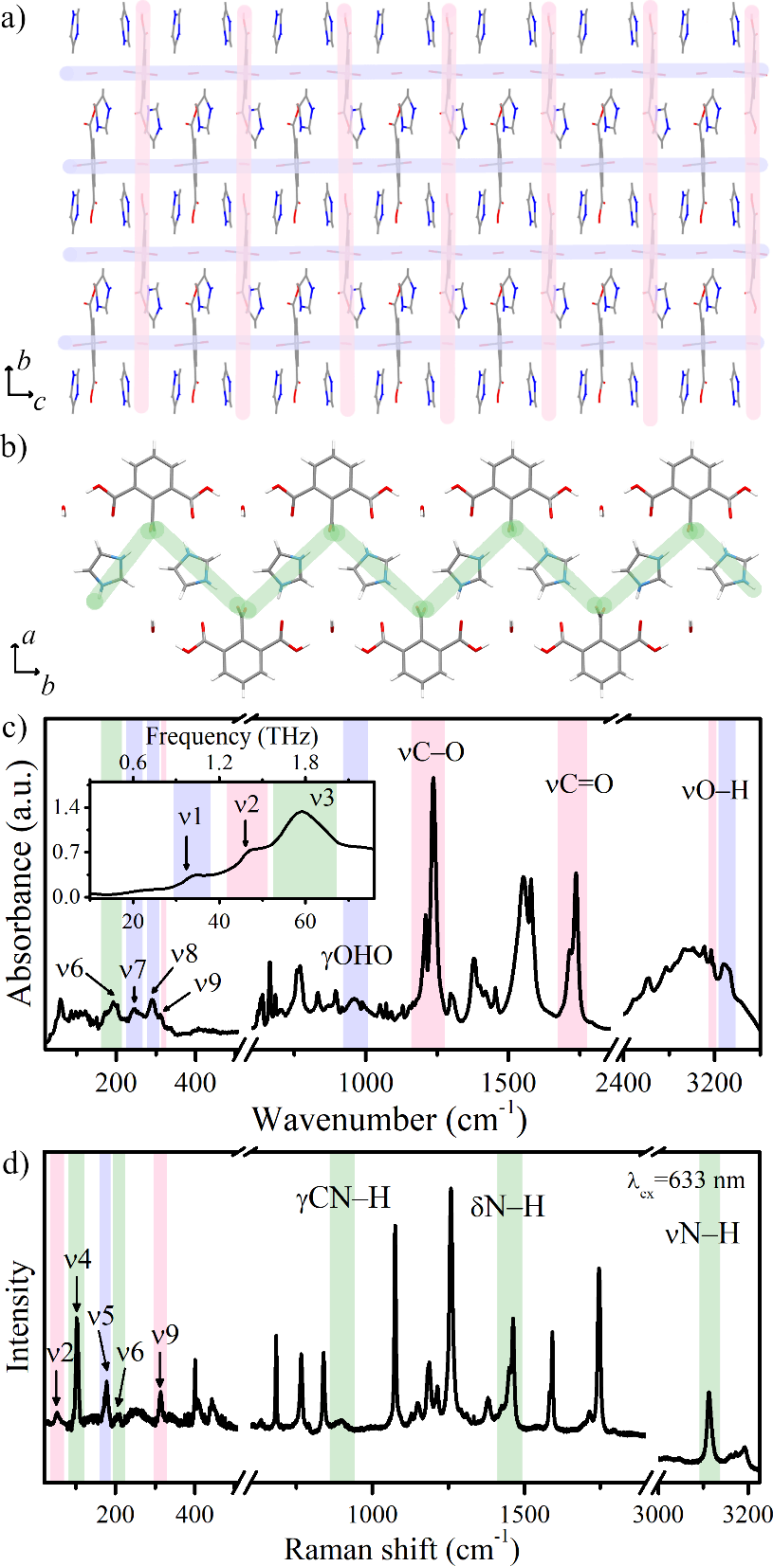
Molecular structure of HemImi·H_2_O with marked O–H···O^–^ (blue) and O–H···O (pink) HBs described by the *C*_2_^2^(5) (water is a H donor) and the *C*_2_^2^(9) (anion is a H donor), respectively (a); Molecular structure with marked N^+^–H···O^–^ HBs (green) forming the *C*_2_^2^(8) chain motif (b). The FT-IR spectrum in the THz, FIR and MIR ranges (c) and the Raman spectrum (d) with marked bands characteristic of water, acid ion and the imidazole ion. The HB motifs are described according to the method proposed by Etter.^31^

### O–H···O^–^ HBs

They connect water molecules with carboxylate groups (water molecules as a H donor), forming a *C*_2_^2^(5) chain motif in the [001] direction (see Figure 1a, blue color).^30^ Based on the energy,^32^ there occur two different HBs: O16–H1···O9b (E_HB_ = −4.85 kcal·mol^–1^) and O16–H2···O8b (E_HB_ = −10.04 kcal·mol^–1^),^30^ which are of medium strength. Water has characteristic infrared bands (see Figure 1c) which are not observed in the Raman spectrum.^33^ The broad band at 3309 cm^–1^ is related to the stretching vibration of O–H and medium-intensity band at 951 cm^–1^ to the out-of-plane bending of the O–H group (from the carboxyl group): γOHO^–^.^30,34^ In the FIR and THz regions, we also observed characteristic phonon bands of O–H···O^–^ HBs at 291 (ν_8_), 246 (ν_7_), 178 (ν_5_, Raman), and 34 (ν_1_) cm^–1^ (Tab. S1).^34^

### O–H···O HBs

They connect carboxyl groups in anions with water molecules (acid ion acts as a H donor) to form chain motifs *C*_2_^2^(9) in the [010] direction (see Figure 1a, pink color).^30^ Carboxyl groups are connected to water via two energetically different HBs: O12b–H12b···016 (E_HB_ = −10.66 kcal·mol^–1^) and 015b–H15b···016 (E_HB_ = −8.32 kcal·mol^–1^).^30^ In terms of energy, these bonds are of medium-strength. Carboxylic groups of anions have characteristic bands in FTIR and Raman spectra.^35^ Bands related to the symmetric and asymmetric stretching vibration of the O–H bond in COOH occur at 3190 and 3170 cm^–1^ in FTIR spectra, respectively (Figure 1c). There are two bands at 1737 and 1715 cm^–1^ connected with C=O stretching. The very intense band at 1237 cm^–1^ is related to the stretching vibration of the C–O bond in COOH groups. In the lattice vibrations region, phonon modes related to O–H···O HBs network can be found at 312 (ν_9_), and 47 (ν_2_) cm^–1^.

### N^+^–H···O^–^ HBs

They connect cations with carboxylate groups (cations as a H donor), creating a *C*_2_^2^(8) chain motif along the [010] direction (Figure 1b, green color).^30^ We can distinguish two energetically different N^+^–H···O^–^ HBs: N1a–H1a···O8b (E_HB_ = −7.14 kcal·mol^–1^, medium strength HB) and N2a–H2a···O8b (E_HB_ = −1.71 kcal·mol^–1^, weak).^30^ In the Raman and FTIR spectra, bands associated with N^+^–H···O^–^ are visible. Bands associated with N–H stretching vibrations occur at 3112 and 3159 cm^–1^ (Figure 1d). Bands associated with out-of-plane and in-plane deformation vibrations occur at 896 and 634 cm^–1^, respectively. The bands associated with the asymmetric and symmetric stretching vibrations of the COO^–^ group occur at 1553 and 1378 cm^–1^, respectively. The 1462 cm^–1^ band is associated with νC–N + δN–H vibrations. In addition, within the phonon vibration range, relevant bands are found at 192 (ν_6_), 102 (ν_4_, Raman), 59 (ν_3_) cm^–1^.

The arrangement of the ions within the crystal structure, along with their N^+^–H···O^–^ HBs, leads to the formation of an approximately helical HB structure. The helix parameters such as semi-major axis (a_h_) and semi-minor axis (b_h_), and helix-pitch (S_h_) are equal to 1.59, 1.26, and 11.74 Å, respectively (see Figure S2).

## Temperature-Dependent Study

The behavior of HB networks determines the thermal properties of salts obtained from carboxylic acids and heterocyclic molecules.^36–38^ For this reason, the analysis of temperature changes in the number of bands and their parameters in HemImi·H_2_O focuses on the bands associated with HBs. The bands characterized by an anharmonic shape were fitted using the SplitPearson7 model in the Fityk program,^39^ which allows the determination of parameters such as a (low-width-at-half-maximum) and b (high-width-at-half-maximum, see Figure S3a), as well as the intensity and full width at half-maximum (Δν). The anharmonicity parameter was determined using the formula:^40,41^

$$\Psi \left(A\right)=1+\left(\frac{1}{\pi }\right)\left[1-\left(\frac{b}{a}\right)\right]\;\mathrm{and}\;\Psi \left(A\right)=1-\left(\frac{1}{\pi }\right)\left[1-\left(\frac{a}{b}\right)\right]$$

for hard (*a* > *b*) and soft (*a* < *b*) anharmonic force characteristic, respectively. Furthermore, the damping parameter was calculated based on the Δν of the bands using the relationship Δν/ν_0_, where ν_0_ denotes the peak frequency.

In Figure 2a,b we observe the temperature evolution of the modes in the range from 3000 to 3410 cm^–1^; Figure 2a shows the infrared active modes and Figure 2b the Raman active modes (see Figures S4 and S5 for the raw spectra). At 393 K, three broad bands are observed at 3309 (νOH in H_2_O), 3166 (νOH in COOH), and 3113 cm^–1^ (νNH). They are characterized by an anharmonic shape (Figure 2c-e shows the temperature dependence of their anharmonicity coefficient; Figure S3b shows an example of fitting of FTIR spectrum). Reducing the temperature to 293 K results in the splitting of the 3309 and 3166 cm^–1^ bands into two components at 3275, 3340 cm^–1^ (blue) and 3171, 3192 cm^–1^ (pink), respectively (see Figure 2a). As the temperature decreases, the position of the 3275 cm^–1^ band shifts quite significantly toward a lower wavenumber (red shift), while the position of the 3340 cm^–1^ band toward a higher wavenumber (blue shift). Another band splitting is observed near 150 K for the stretching of the O–H bond (blue) and N–H bond (green), Figure 2a. Both components of the N–H bond stretching red shift as the temperature decreases, indicating an elongation of the N–H bond and thus a shortening of the N^+^–H···O^–^ HB.^38^ In the Raman spectra, we observe bands associated with the stretching of O–H bonds in COOH groups (pink) and of N–H bonds (green) (see Figure 2b). We observe a band splitting near 200 K in both these systems of bands; the N–H bond related bands start to soften (red shift) below 150 K.

**Figure 2:**
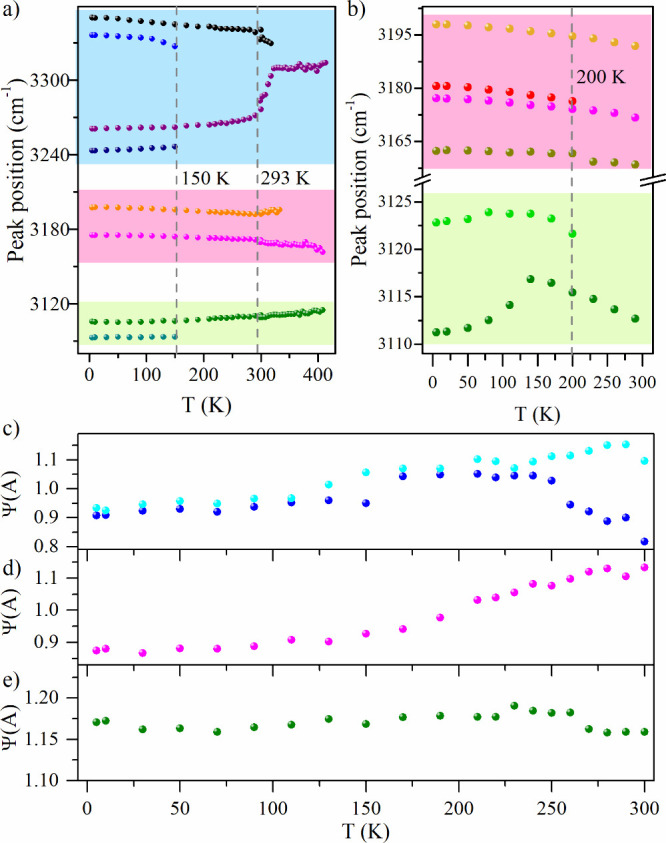
Temperature dependence of the FTIR (a) and Raman (b) band positions of HemImi·H_2_O in the range of 3080–3359 cm^–1^ and 3110–3200 cm^–1^, respectively; the anharmonicity coefficients of the bands: 3275 (light blue) and 3340 (dark blue, c); 3167 (d); 3113 cm^–1^ (e). The background colors maintain the color scheme introduced in Figure 1 for the bands related to various HBs (O–H···O^–^ HBs are marked blue, O–H···O pink, and N^+^–H···O^–^ green).

The temperature dependence of selected IR spectra in the range of 725–1010 cm^–1^ as a function of temperature from 5 to 393 K, together with the temperature dependence of the band positions, is shown in Figure 3. A broad band near 951 cm^–1^ is related to O–H···O^–^ HBs as explained in the previous paragraph; its intensity dramatically increases with decreasing temperature: indeed, it is practically not detected at the highest temperatures while it becomes well pronounced below the 200 K (see Figure S6). In the following we focus on lower-frequency bands (yellow background in Figure 3a) and, as can be seen in Figure 3b, the number of bands in the analyzed range changes with temperature. Above 293 K (Phase I in Figure 3b), six bands can be distinguished: 766, 771, 832, 872, 893, and 916 cm^–1^. These bands are broad, suggesting a disorder (i.e., undefined position and/or orientation of the rotators). Below the room temperature, an additional band appears at 883 cm^–1^. Below 150 K, one finds up to 15 bands in the analyzed spectral range (see Figure 3b). The bands at 872, 831, and 760 cm^–1^ are associated with δCOO + δCCC, γC–CH + γCOOH, and δCC–H + δCOO (related to anion dynamics, Table S1), respectively. As the temperature further decreases, the position of these bands changes slightly, but no additional components appear. The bands at 916, 893, and 771 cm^–1^ are associated with δCNC, γCNH, and γNCH (cation dynamics), respectively. The Raman spectra in this range (Figure S7) show qualitatively very similar behavior, i.e., mode splitting is observed at similar temperatures. In the spectrum calculated for an isolated part of the structure involving two molecules of acid and imidazolium ions (see the scheme in Figure S8) using DFT methods, a similar number of bands is observed as in the spectrum recorded at 5K (Figure 3a).

**Figure 3:**
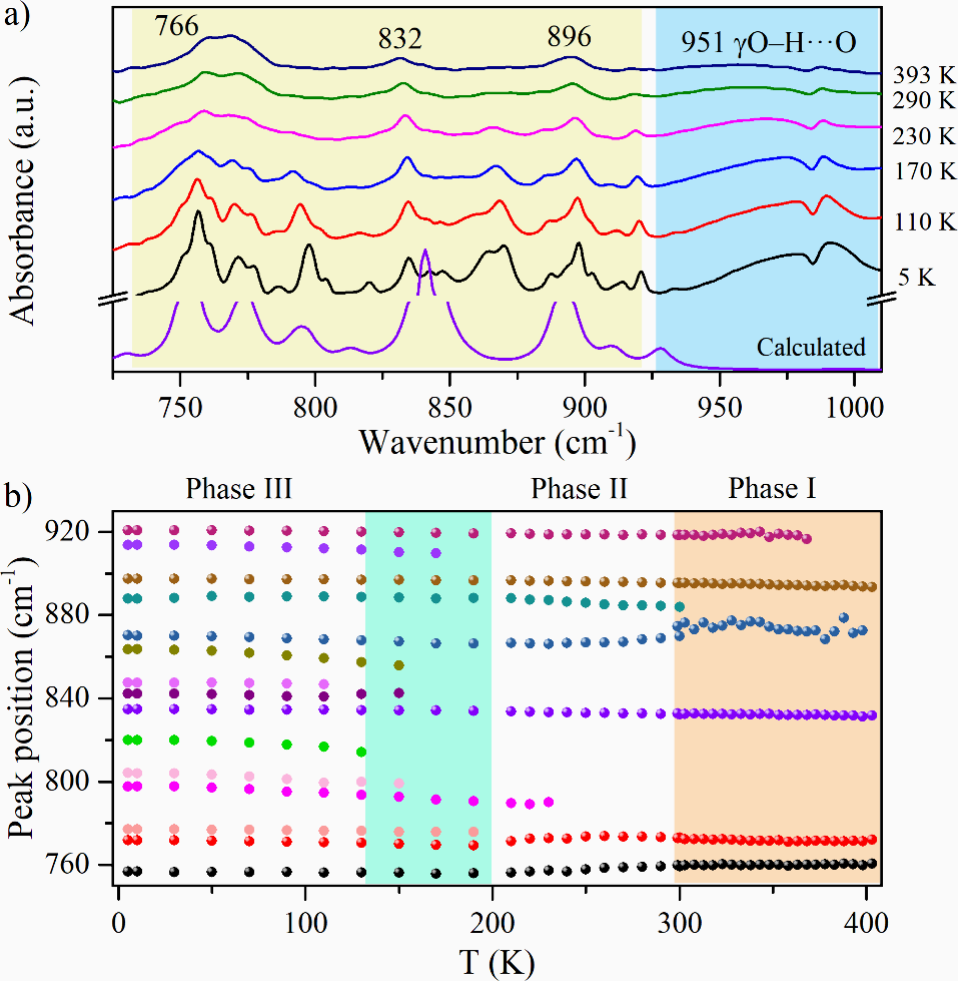
Temperature evolution of experimental and calculated FTIR spectra in the range of 730–1030 cm^–1^ (a); Temperature dependence of band positions, with highlighted areas showing the occurrence of phases I–III. The green area represents the transition between the disordered and ordered phases II and III (b). A scaling factor of 0.99 for frequency was applied to the calculated spectrum. The model system for which the IR spectrum was calculated is shown in Figure S8.

Within the range of phonon vibrations, at room temperature, we observe broad bands of low intensity (see Figures S9–S11). As the temperature decreases, their intensity increases and their half-width decreases. Additionally, below 200 K, new bands appear at 137 and 130 cm^–1^ (Figure S9b), as well as at 41 and 47 cm^–1^ below 125 K (Figure S10).

## Phase Transition from Phase I to Phase II

A very broad peak related to νO–H originating from water is observed near 3300 cm^–1^ (Figures 1c, 2a, S4) with an full width at half maximum (fwhm) of 73 cm^–1^ at 393 K (i.e., it shows a nonvanishing intensity nearly over the whole range marked in blue in Figure 2a). This indicates a high dynamical disorder of the water molecules with respect both to their orientation and to their position within the system. This finding is also confirmed by the anharmonic shape of this band expressed by the anharmonicity parameter Ψ plotted in Figure 2c. As the temperature decreases, this band shifts toward a lower wavenumber (Figure S12) indicates a shortening of the O–H···O^–^ HBs and, consequently, an elongation of the O–H bond. Below the room temperature, the fwhm of the band is significantly decreased (Figure S12) and two components appear in the spectrum instead of a single one. Above the room temperature, water molecules constitute a dynamic phase where the short-range order is absent. Only long-range order occurs, as in plastic materials, but with well-defined motion.^4,42^ The disordered position of water molecules causes the length of O–H···O^–^ HBs to change (shortening and lengthening), and increases the probability of hydrogen jumping within HBs. This creates the possibility of forming hydronium ions, which are important because of the material’s conductive properties.^28^

The thermal behavior of the 951 cm^–1^ band associated with γOHO^–^ shown in Figure 3a also confirms the dynamically disordered phase of the water molecule subnetwork. Since both νO–H and γOHO^–^ bands still evolve quite significantly below the room temperature we think that the disorder is only partially lifted upon a temperature decrease to ∼ 270 K. The observed changes thus indicate that, close to the room temperature, we observe a transition from positional to orientational disorder of water molecules (Figure 4a).

**Figure 4:**
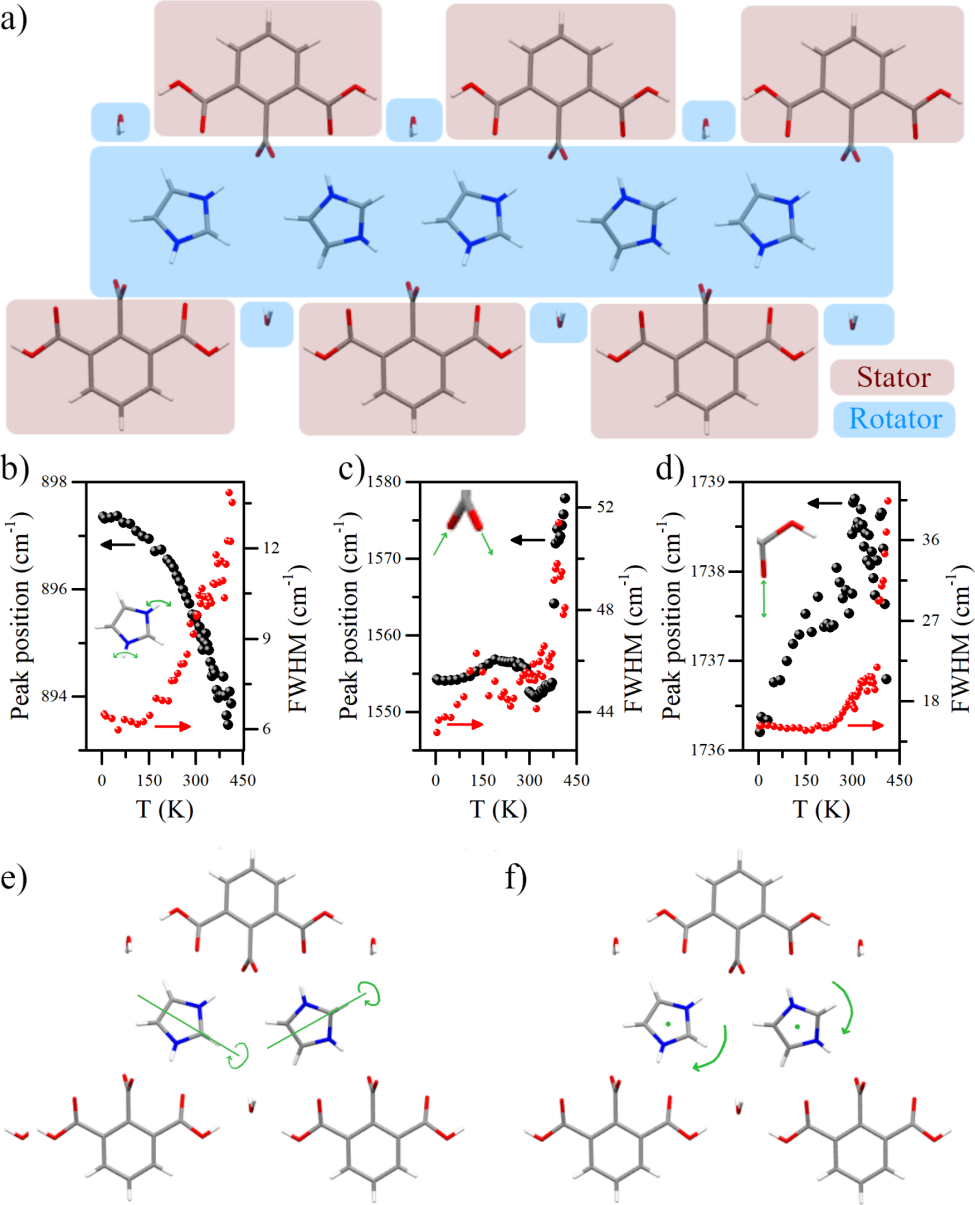
Crystal structure of HemImi·H_2_O divided into static (stator) and dynamic (rotator) parts of the molecular system above 200 K (a); temperature dependence of the peak position and fwhm of the bands associated with γCNH (b), νCOO (c) and νC=O (d). Possible mechanisms of cation dynamics: rotation around an axis passing through the C1–H bond (e), libration around the center of the imidazole ring (green dot, f).

The presented hypothesis is confirmed by the thermal analysis of the following bands: 893 cm^–1^ (deformation of γCNH bond), 1554 cm^–1^ (stretching of COO bonds), and 1710 and 1736 cm^–1^ (both related to the stretching of C=O bonds), see Table S1 and Figure 4b–d. The fwhm of γCNH band is quite large at high temperatures and significantly decreases upon temperature decrease, indicating a decreasing disorder; however, no anomaly is observed near 270 K, indicating that imidazole is not involved in the ordering process observed close to the room temperature, i.e., it stays in the rotator phase (see Figure 4b). The temperature dependence of the position of the COO stretching shows a minimum near the room temperature (Figure 4c) connected with the ordering of water molecules. Stabilizing the position of the water molecule causes C=O bonds in COOHb and COOHd groups to shorten and lengthen, respectively (see Figure S1). Consequently, upon cooling below the room temperature, a significant variation in the positions of the 1710 and 1737 cm^–1^ bands is observed (see Figures 4d and S13). Below, the fwhm decreases and the position becomes more defined. This analysis revealed that, in the disordered phase, water influences the dynamics of both O–H···O^–^ and O–H···O HBs. Consequently, it modifies the hydrogen dynamics in the *b* and *c* directions of the unit cell.

## Phase Transition from Phase II to Phase III

The number of bands increases significantly upon cooling in the temperature range 200 – 150 K both in the Raman and IR spectra. Namely, in the Raman spectrum, splitting of the 3112 (associated with νNH) and 3171 cm^–1^ (associated with νOH in COOH) bands is observed (Figure S5). The damping coefficient of the OH stretching mode (3171 cm^–1^) dramatically decreases upon cooling at higher temperatures but this trend stops in the 200–150 K temperature range and below, the fwhm of this mode remains nearly constant (Figures S14, S15). Furthermore, the number of IR bands in the 650–1000 cm^–1^ increases from 6 to 15 on cooling below 150 K (Figure 3). All these observations indicate another ordering process to occur which concerns mainly a freezing of the rotation of imidazole and water molecules. As a result of this transition, the rotation of imidazole ions (heavier) and water (lighter) ceases at different temperatures. Imidazole ions become static first near 200 K and, progressively, the water stops the rotations upon further cooling (down to 150 K).

The transition from the dynamic to the static phase of the cations is also indicated by two new bands appearing at 137 and 130 cm^–1^ below 200 K in the Raman spectra associated with N^+^–H···O^–^ HBs (Figure S9b). Similarly, additional bands appear in the Raman spectrum at around 750–780 cm^–1^ (Figure S7), which are associated with deformation vibrations of the N–H bonds. Above 200 K, only the broad bands associated with HBs are observed due to the cation dynamics. At temperatures where the cations in the crystal lattice become ordered, the bands associated with the dynamics of N–H and C–H bonds of varying lengths appear in the spectra as distinct peaks. Analysis of the 893 cm^–1^ band associated with γCNH showed that its position and fwhm change drastically with decreasing temperature down to 200 K (see Figure 4b). Below 200 K, these parameters change only slightly, which confirms the ordering of the cations at temperatures below 200 K.

In the THz range, two new bands appear below 125 K in the range of 40–51 cm^–1^ (Figure S10), which are also related to the dynamics of the N^+^–H···O^–^ HB network. Structural disorder of cations was previously observed in the group of heterocyclic salts with carboxylic acids in imidazolium malonate,^20–22^ imidazole succinate, and imidazole glutarate.^23^ However, unlike in HemImi·H_2_O, the cation in imidazolium malonate is not dynamic and can only occupy two equivalent positions with equal probability. Furthermore, lowering the temperature does not lead to an ordered structure. However, none of the above compounds exhibited a dynamic phase of cation sublattice, as observed in HemImi·H_2_O.

Theoretical calculations concerning possible movements of the imidazole ion in the base subnetwork were performed using DFT methods. Figure S16 shows model systems for the PES calculations for the libration (a) and rotation (b) motions of the cation in the vicinity of the nearest coordination zone (cf. also Figure 4 e,f). Potential barriers have been identified in the case of libration. For 1L, 2L, and 3L, the energies are approximately 0.29–0.30 eV, 0.12 eV, and 0.375–0.385 eV, respectively (see Figure S17 and Table S2). In the case of imidazole ion rotation, two barriers with similar energies of approximately 6.39–6.55 eV were observed. The rotational motion of the imidazole is hindered by the considerable amount of energy required to reach the position at nearly 90° with respect to the in-plane position (0° is the crystal structure position of an ion). However, in the case of imidazole ion libration, the energy values of around 0.1 eV indicate the possibility of such a movement. The calculations suggest that the imidazole ion can act as a rotator within the base subnetwork, performing librational motion within the plane of the molecule (the activation energy is about 15 times lower than for rotation, see Figure 4f).

## Proton–Phonon Coupling

The temperature dependences of the bands associated with phonons ν_4_ and ν_5_ are very similar to each other below 200 K (Figure 5). The ν_4_ band is linked predominantly with γNHO, and ν_5_ with δOHO^–^. The observed temperature dependences were modeled using the Boltzmann equation:

$$\nu_{0}\left(T\right)=A_{2}+\frac{\left(A_{1}-A_{2}\right)}{1+\exp\left(\frac{T-T_{0}}{dT}\right)}$$

where *A*_1_ and *A*_2_ are, respectively, the initial and final value of peak position, *T*_0_ determines the center of the function, and *dT* is a temperature constant characterizing the speed of the variation between the initial and final value. The temperature dependence of the ν_4_ and ν_5_ bands is highly similar. This is particularly evident in the *T*_0_ and *dT* parameter values, which are 97.0 ± 2.1 K and 19.7 ± 1.9 K for ν_4_, and 97.7 ± 1.6 K and 19.2 ± 1.4 K for ν_5_, respectively. The similarity in the temperature dependence of the position of these bands suggests that the same processes are responsible for the changes. Furthermore, they suggest the presence of coupling between these phonon excitations.

**Figure 5:**
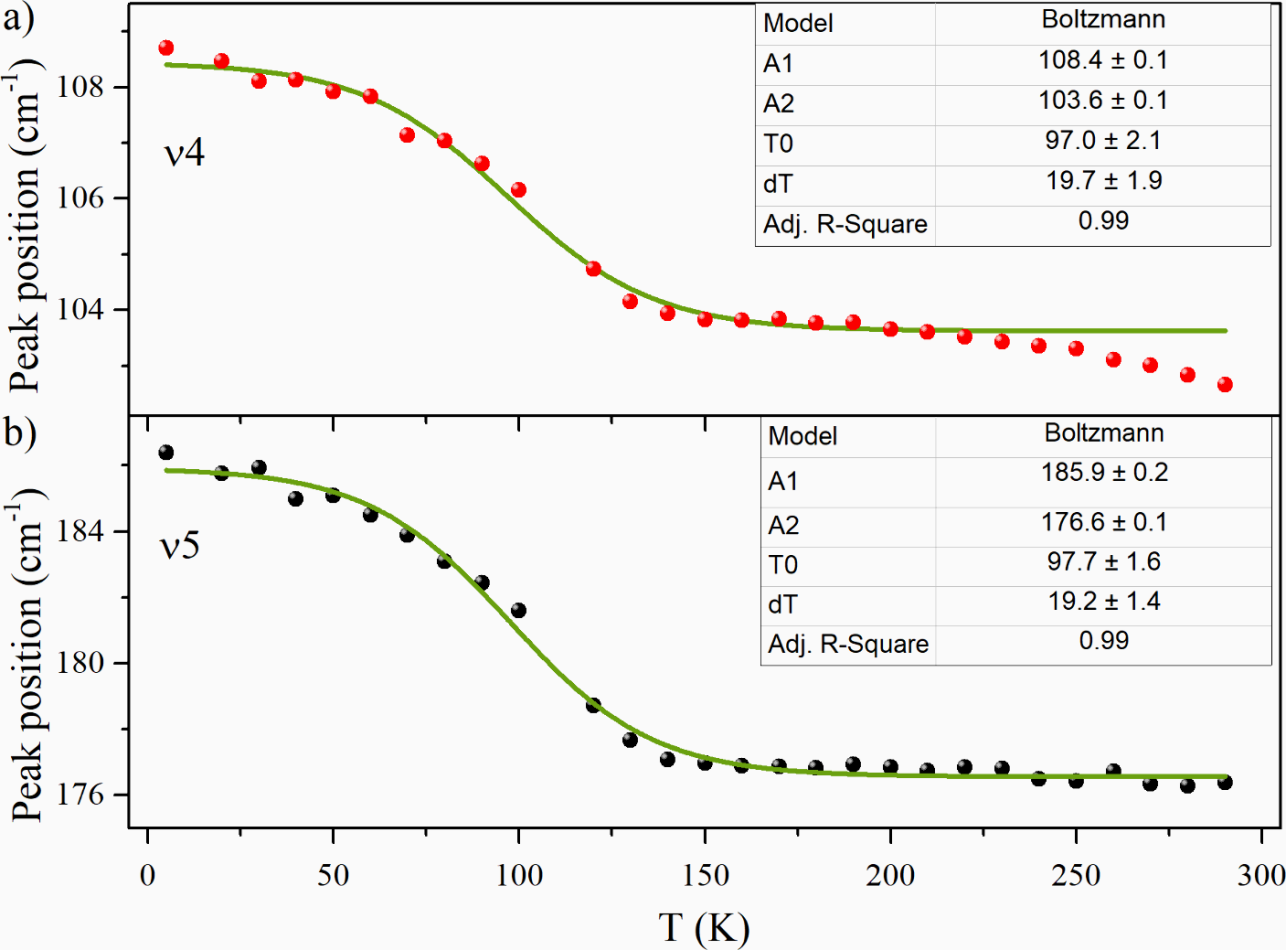
Temperature dependence of the position of ν4 (a) and ν5 (b) bands, together with the fitted Boltzmann model.

Analysis of lattice vibrations ν_4_ and ν_5_ revealed that they exhibit either an ordered or a disordered phase. Positional and orientational disorder are treated in the same way and there is no difference between them. The Boltzmann model therefore enables us to distinguish between disordered and ordered phases, the latter of which involve HBs coupled in different directions, as well as the temperature range in which the order–disorder transition occurs.

In HemImi·H_2_O, phonons generated by the vibration of N^+^–H···O^–^ and protons in O–H···O and O–H···O^–^ HBs are coupled. The contribution of N^+^–H···O^–^ HBs to the coupling can be seen from the thermal behavior of the 3112 cm^–1^ band, which is linked to the stretching of the N–H bond (see Figure S15). At 150 K, the band splits into two components, and below 100 K, the half-width of both components changes only slightly. The anharmonicity coefficient is around 1.17, indicating hard anharmonicity (i.e., the vibration only deforms the electron cloud).

The bands associated with the νOH vibration in the O–H···O^–^ HBs at 3301 and 3327 cm^–1^ split at 150 K (see Figure S12). The anharmonicity coefficient of these bands changes in different ways as the temperature decreases below 300 K, finally settling at a value of approximately 0.9 below 140 K (see Figure 2c). Similar thermal behavior and a value of approximately 0.9 below 140 K were observed for the 3166 cm^–1^ band (associated with the νOH vibration in the O–H···O HBs, Figure 2d). A value of 0.9 for the anharmonicity coefficient indicates soft anharmonicity, i.e. a change in the shape of the electron cloud of the H acceptor.^40^ Similar behavior of the fwhm as a function of temperature is also observed for the 3301, 3327, and 3166 cm^–1^ bands (Figures S12b and S14b). When the temperature is lowered from 300 to 140 K, the fwhm decreases and below 140 K, it keeps an approximately constant level.

The contribution of O–H···O^–^ and O–H···O HBs to the coupling is also evident in the strongly anharmonic shape of the IR band 951 cm^–1^ associated with γOHO (see Figures 3a and S6). Coupling effects typically cause an asymmetric line shape. In addition, this band has a characteristic shape that reflects the average HB strength. This strength is influenced by two mechanisms: anharmonic coupling and Fermi resonance between the |01> and |02> states.^43^ The intensity of this band increases with decreasing temperature and extends from 930 to 1020 cm^–1^ (see Figure 3a). Analysis of the 1580 cm^–1^ band also revealed an anharmonic shape (see Figure S13). Its position remains fixed below 140 K (equivalent to 1554 cm^–1^, see Figure 4c). Furthermore, this band is broad, with an fwhm of 44 cm^–1^ below 140 K.

To sum up, the paper presents the vibrational dynamics in hydrated imidazole hemimelitate, which is the first amphidynamic molecular crystal in the group of salts obtained on the basis of imidazole and aromatic carboxylic acids. The studied compound contains a static subnetwork formed by acid ions and a dynamic subnetwork consisting of cations and water molecules. The formation of weak and medium-strength hydrogen bonds, which create a unique helical shape, meets the conditions for obtaining a dynamic phase at high temperatures. We detected a transition from positional to orientational disorder of water molecules upon the sample cooling close to the room temperature. The orientational disorder of imidazole and water molecules then progressively disappears below 200 K and the sample becomes completely ordered near 150 K. Our experiments also reveal that this salt exhibits a proton-phonon coupling below 100 K.

Our analysis allows us to make progress in designing new amphidynamic materials. We show that combining a heterocyclic molecule, such as imidazole, with a sufficiently large acid, such as hemimelitic acid, enables the formation of channels through which dynamic phase movement is possible.

## Supplementary Material



## Notes

Experimental details, including the synthesis procedure and analyses of the HB networks by means of DFT and PES. Experimental and theoretical analyses of the band positions. Analysis of band parameters under temperature (PDF)

## References

[1] Asher M., Bardini M., Catalano L., Jouclas R., Schweicher G., Liu J., Korobko R., Cohen A., Geerts Y., Beljonne D., Yaffe O. (2023). Mechanistic View on the Order–Disorder Phase Transition in
Amphidynamic Crystals. J. Phys. Chem. Lett..

[2] Colin-Molina A., Karothu D. P., Jellen M. J., Toscano R. A., Garcia-Garibay M. A., Naumov P., Rodríguez-Molina B. (2019). Thermosalient Amphidynamic
Molecular Machines: Motion at the Molecular and Macroscopic Scales. Matter.

[3] Kottas G. S., Clarke L. I., Horinek D., Michl J. (2005). Artificial
Molecular
Rotors. Chem. Rev..

[4] Liepuoniute I., Jellen M. J., Garcia-Garibay M. A. (2020). Correlated
Motion and Mechanical
Gearing in Amphidynamic Crystalline Molecular Machines. Chem. Sci..

[5] Comotti A., Bracco S., Yamamoto A., Beretta M., Hirukawa T., Tohnai N., Miyata M., Sozzani P. (2014). Engineering Switchable
Rotors in Molecular Crystals with Open Porosity. J. Am. Chem. Soc..

[6] Catalano L., Naumov P. (2018). Exploiting Rotational Motion in Molecular Crystals. CrystEngComm.

[7] Garcia-Garibay M. A. (2005). Crystalline
Molecular Machines: Encoding Supramolecular Dynamics into Molecular
Structure. Proc. Natl. Acad. Sci. U.S.A..

[8] Sato O. (2016). Dynamic Molecular
Crystals with Switchable Physical Properties. Nat. Chem..

[9] Roy I., Stoddart J. F. (2019). Amphidynamic Crystals Key to Artificial Molecular Machines. Trends Chem..

[10] Zafar Z., Zafar A., Guo X., Lin Q., Yu Y. (2019). Raman Evolution
of Order–Disorder Phase Transition in Multiaxial Molecular
Ferroelectric Thin Film. J. Raman Spectrosc..

[11] Liu M., Hao X., Hu Y., Zhang W., Shi P. (2025). The Tuning of Structure
Types and Switchable Dielectric Behavior in Pyridine Bromocuprate
(II) Complexes. Eur. J. Inorg. Chem..

[12] Lindgren J., Olbert-Majkut A., Pettersson M., Kiljunen T. (2012). Librational Motion
of CO in Solid Ar: Raman and IR Spectra and Quantum Simulations. Low Temp. Phys..

[13] Zhang Z., Gong W., Zhao X., Qi J., Li M., Torii S., Chen H., Yin W., Ohara K., Zhang Z., Kawakita Y., Li B. (2023). Local Atomic
Structures
and Lattice Dynamics of Inverse Colossal Barocaloric Ammonium Thiocyanate. Phys. Rev. Mater..

[14] Sato C., Dekura S., Sato H., Sambe K., Takeda T., Kurihara T., Mizuno M., Taniguchi T., Wu J., Nakamura T., Akutagawa T. (2024). Proton Conduction
in Chiral Molecular
Assemblies of Azolium–Camphorsulfonate Salts. J. Am. Chem. Soc..

[15] Hoshino N., Akutagawa T. (2022). Thermal Conductivities
and Figures of Merit of Tetracyanoquinodimethane-Based
Thermoelectric Materials Consisting of Cations Exhibiting Order–Disorder
Transitions. Cryst. Growth Des..

[16] Besara T., Jain P., Dalal N. S., Kuhns P. L., Reyes A. P., Kroto H. W., Cheetham A. K. (2011). Mechanism of the Order–Disorder
Phase Transition, and Glassy Behavior in the Metal-Organic Framework
[(CH_3_)_2_NH_2_]­Zn­(HCOO)_3_. Proc. Natl. Acad. Sci. U.S.A..

[17] Lv Y., Liang J., Xiong Z., Yang X., Li Y., Zhang H., Xiang S., Chen B., Zhang Z. (2024). Smart-Responsive
HOF Heterostructures with Multiple Spatial-Resolved Emission Modes
toward Photonic Security Platform. Adv. Mater..

[18] Huang Y., Gottfried J. L., Sarkar A., Zhang G., Lin H., Ren S. (2023). Proton-Controlled
Molecular Ionic Ferroelectrics. Nat. Commun..

[19] Matsui H., Shimatani K., Ikemoto Y., Sasaki T., Matsuo Y. (2020). Phonon-Assisted
Proton Tunneling in the Hydrogen-Bonded Dimeric Selenates of Cs3H­(SeO4)­2. J. Chem. Phys..

[20] Ławniczak P., Pogorzelec-Glaser K., Pietraszko A., Hilczer B. (2021). Effect of Disordered
Imidazole Substructure on Proton Dynamics in Imidazolium Malonic Acid
Salt. Acta Crystallogr. B Struct Sci. Cryst.
Eng. Mater..

[21] Ławniczak P., Pogorzelec-Glaser K., Pietraszko A., Hilczer B. (2017). Impedance Spectroscopy
Studies of Proton Conductivity in Imidazolium Malonate. Solid State Ionics.

[22] Callear S. K., Hursthouse M. B., Threlfall T. L. (2010). A Systematic
Study of the Crystallisation
Products of a Series of Dicarboxylic Acids with Imidazole Derivatives. CrystEngComm.

[23] Sunairi Y., Dekura S., Ueda A., Ida T., Mizuno M., Mori H. (2020). Anhydrous Purely Organic Solid-State Proton Conductors: Effects of
Molecular Dynamics on the Proton Conductivity of Imidazolium Hydrogen
Dicarboxylates. J. Phys. Soc. Jpn..

[24] Ivanovska T., Quarti C., Grancini G., Petrozza A., De Angelis F., Milani A., Ruani G. (2016). Vibrational
Response of Methylammonium
Lead Iodide: From Cation Dynamics to Phonon–Phonon Interactions. ChemSusChem.

[25] Bajaj N., Bhatt H., Vishwakarma S. R., Deo M. N. (2019). Orientational Adaptations
Leading to Plausible Phase Transitions in l -Leucine at Low Temperatures:
Revealed by Infrared Spectroscopy. J. Phys.
Chem. B.

[26] Hetmańczyk J., Hetmańczyk Ł., Migdał-Mikuli A., Mikuli E., Wesełucha-Birczyńska A. (2012). Raman Light
Scattering, Infrared Absorption and DSC Studies of the Phase Transition
and Vibrational and Reorientational Dynamics of H_2_O Ligands
and ClO_4_
^–^ Anions in [Ba­(H_2_O)_3_]­(ClO_4_)_2_. J. Raman Spectrosc..

[27] Takehara H., Nagai M., Ashida M., Okuyama Y., Kani Y. (2023). Phonons Damped
by Proton Doping in Barium Zirconate. J. Phys.
Chem. C.

[28] Matsui H., Takebe Y., Takahashi M., Ikemoto Y., Matsuo Y. (2024). Proton Transfer
Driven by the Fluctuation of Water Molecules in Chitin Film. J. Chem. Phys..

[29] Matsui H., Fukuda K., Takano S., Ikemoto Y., Sasaki T., Matsuo Y. (2022). Mechanisms of the Antiferro-Electric Ordering in Superprotonic
Conductors Cs_3_H­(SeO_4_)_2_ and Cs_3_D­(SeO_4_)_2_. J. Chem.
Phys..

[30] Zięba S., Mizera A., Markiewicz K. H., Dubis A. T., Ławniczak P., Gzella A., Siergiejczyk L., Łapiński A. (2023). Effect of
Azole Counterions on Thermal and Transport Properties of the Hydrated
Salts of Hemimelitic Acid. J. Phys. Chem. C.

[31] Etter M. C. (1991). Hydrogen
Bonds as Design Elements in Organic Chemistry. J. Phys. Chem..

[32] Espinosa E., Molins E., Lecomte C. (1998). Hydrogen Bond
Strengths Revealed
by Topological Analyses of Experimentally Observed Electron Densities. Chem. Phys. Lett..

[33] Yu C.-C., Chiang K.-Y., Okuno M., Seki T., Ohto T., Yu X., Korepanov V., Hamaguchi H., Bonn M., Hunger J., Nagata Y. (2020). Vibrational
Couplings and Energy Transfer Pathways
of Water’s Bending Mode. Nat. Commun..

[34] Flór M., Wilkins D. M., De La
Puente M., Laage D., Cassone G., Hassanali A., Roke S. (2024). Dissecting the Hydrogen Bond Network
of Water: Charge Transfer and Nuclear Quantum Effects. Science.

[35] Zięba S., Piotrowska A., Mizera A., Ławniczak P., Markiewicz K. H., Gzella A., Dubis A. T., Łapiński A. (2021). Spectroscopic
and Structural Study of a New Conducting Pyrazolium Salt. Molecules.

[36] Zięba S., Gzella A., Dubis A. T., Łapiński A. (2021). Combination
of Negative, Positive, and Near-Zero Thermal Expansion in Bis­(Imidazolium)
Terephthalate with a Helical Hydrogen-Bonded Network. Cryst. Growth Des..

[37] Zięba S., Rusek M., Katrusiak A., Gzella A., Dubis A. T., Łapiński A. (2023). Helical Model
of Compression and
Thermal Expansion. Sci. Rep..

[38] Zięba S., Dubis A. T., Rusek M., Katrusiak A., Gzella A., Łapiński A. (2025). Negative Thermal
Expansion
and Linear Compressibility in 1 *H* -Imidazol-3-Ium
2-Hydroxybenzoate with a Helical Network of Hydrogen Bonds. Phys. Chem. Chem. Phys..

[39] Wojdyr M. (2010). Fityk: A General-Purpose
Peak Fitting Program. J. Appl. Crystallogr..

[40] Plendl J. N. (1961). Some New
Interrelations in the Properties of Solids Based on Anharmonic Cohesive
Forces. Phys. Rev..

[41] Plendl J. N., Mansur L. C. (1972). Anomalous Thermal
Expansion with Infrared Spectroscopy. Appl.
Opt..

[42] Das S., Mondal A., Reddy C. M. (2020). Harnessing
Molecular Rotations in
Plastic Crystals: A Holistic View for Crystal Engineering of Adaptive
Soft Materials. Chem. Soc. Rev..

[43] Bratos S. (1975). Profiles of
Hydrogen Stretching Ir Bands of Molecules with Hydrogen Bonds: A Stochastic
Theory. I. Weak and Medium Strength Hydrogen Bonds. J. Chem. Phys..

