# Supplementary material for: Amphidynamic Molecular Crystal with Temperature-Controlled Helical Hydrogen-Bonded Network: Proton Dynamics and Order–Disorder Phase Transition
Source: J Phys Chem Lett. 2026 Mar 24;17(14):4041–9. doi: 10.1021/acs.jpclett.6c00289 (PMC13298902; doi:10.1021/acs.jpclett.6c00289)
Supplement: Supplementary file 1 [file jz6c00289_si_001.pdf]

Supplementary materials: Amphidynamic Molecular Crystal with Temperature-Controlled Helical Hydrogen Bonded Network: Proton Dynamics and Order-Disorder Phase Transition

S. Zięba,[1,2] C. Kadlec,[1] S. Priya,[3] Y. Lin,[3] A. Mizera,[2] M. Dressel,[3] P. Kužel[1]

[1] Institute of Physics, Czech Academy of Sciences, Na Slovance 1999/2, 182 00 Prague 8, Czech Republic

[2] Institute of Molecular Physics, Polish Academy of Sciences, M. Smoluchowskiego 17, 60-179 Poznan, Poland

[3] 1. Physikalisches Institut, Universität Stuttgart, Pfaffenwaldring 57, 70569 Stuttgart, Germany

Methods

Fourier-transform infrared (FT-IR) spectra were recorded as a function of temperature using an Equinox 55 FT-IR spectrometer equipped with a Bruker Hyperion 2000 microscope. Spectra were measured within the spectral range from 600 to 7000 cm^{-1} with a spectral resolution of 2 cm^{-1}. The sample was prepared as a KBr pellet (at concentrations of 1:1000 and 1:500 for room temperature and temperature-dependent measurements, respectively). Vibrational spectra were studied as a function of temperature from 293 to 393 K using a Linkam THMS 600 cryostat with temperature stability of 1 K. Raman spectra were recorded using a LabRAM HR 800 UV HORIBA Jobin Yvon spectrometer with a liquid nitrogen-cooled CCD detector. Spectra were recorded using He–Ne laser excitation (λ = 632.8 nm) and a ×20 objective. The spectral resolution was better than 2 cm^{-1}, and the laser power at the sample was less than 1 mW. An Oxford Inst. CF 2102 cryostat was employed to investigate the spectroscopic properties at temperatures from 5 to 300 K.

Far infrared transmission measurements were done employing a Bruker VERTEX 80V FT-IR spectrometer (Beamsplitter: Mylar), equipped with a HYPERION microscope. A helium-cooled bolometer was used as a detector to record spectra in the region from 100 to 700 cm^{-1}, with a resolution of 2 cm^{-1}. For the FIR measurement, the powdered sample was grinded in Nujol mull. A small amount of this sample was sandwiched between 2 layers of mylar films (with Nujol mull), which was used for the measurement. Two films of mylar sandwiching Nujol mull were used as a reference. The obtained transmission spectra were transformed to get absorption spectra. The sample was placed in a commercial Oxford Instruments Helium-cryostat (Microstat He), which can operate from room temperature to 4 K, using polypropylene windows (transparent in the FIR region).

A custom-made spectrometer based on a Ti:sapphire femtosecond laser with an Optistat (Oxford Instruments) cryostat was used to measure the complex sample transmittance, in order to perform time-domain THz spectroscopy at room temperature. The sample was prepared as a powder in a cuvette (thickness 0.2 mm). Reference measurements with an empty cuvette were performed systematically at each temperature value, enabling a reliable determination of the transmittance.

A commercial TeraView TeraPulse 4000 spectrometer was used to measure THz spectra under temperature. The external channel, featuring remote emitter and receiver, was placed within a nitrogen purge chamber to prevent absorption of THz radiation by water vapor in the air. The absorption of the sample was determined by comparing the transmission between the sample on mylar film and the empty reference mylar film, where the transmission was derived from FFT of the time-domain THz pulse. The home-made zero-field ^{4}He cryostat was used. It operated in the temperature range from room temperature to below 10 K (with the capability to reach as low as 1.5 K), with tilted mylar windows for transporting THz radiation in free space. The powdered sample (7 mg) was grinded and pressed to make a pellet (diameter = 5 mm). This pellet was then glued to a mylar film at the edge. An aperture of 4 mm was used for the transmission measurement, with mylar film as a reference of the same dimension. The obtained transmission spectra were transformed to get absorption spectra.

The Gaussian09 computational package was used to perform quantum mechanical calculations.[1] The first coordination sphere of the base was chosen to create a model system that would simulate the libration and rotation of the imidazole ion (see Fig. S16). The axes of rotation of the imidazole ion are shown in Figure S16. Potential Energy Scan (PES) calculations were performed using DFT methods. The calculations were

performed using the B3LYP,[2–4] cam-B3LYP and ωB97x-D functional,[5,6] with the Pople's 6-311G(d, p) basis set.[7] Calculations were also performed to optimize the geometry and vibrational structure for a model composed of hemimelitate acids and imidazole (see Fig. S8). Calculations were performed for the ionized system (-1) using the ωB97x-D/6-311G(d,p) level of theory. The results of vibrational structure calculations did not show any negative frequencies, which indicates that a local minimum has been reached.

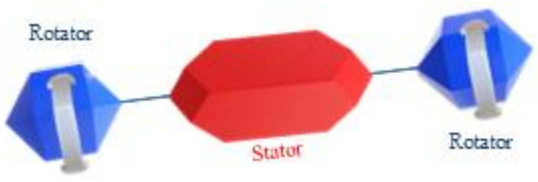

Scheme S1. A schematic presentation of amphidynamic organic materials containing rigid (stator) and dynamic (rotator) elements. The stator and rotator can be connected by either a covalent or a hydrogen bond.

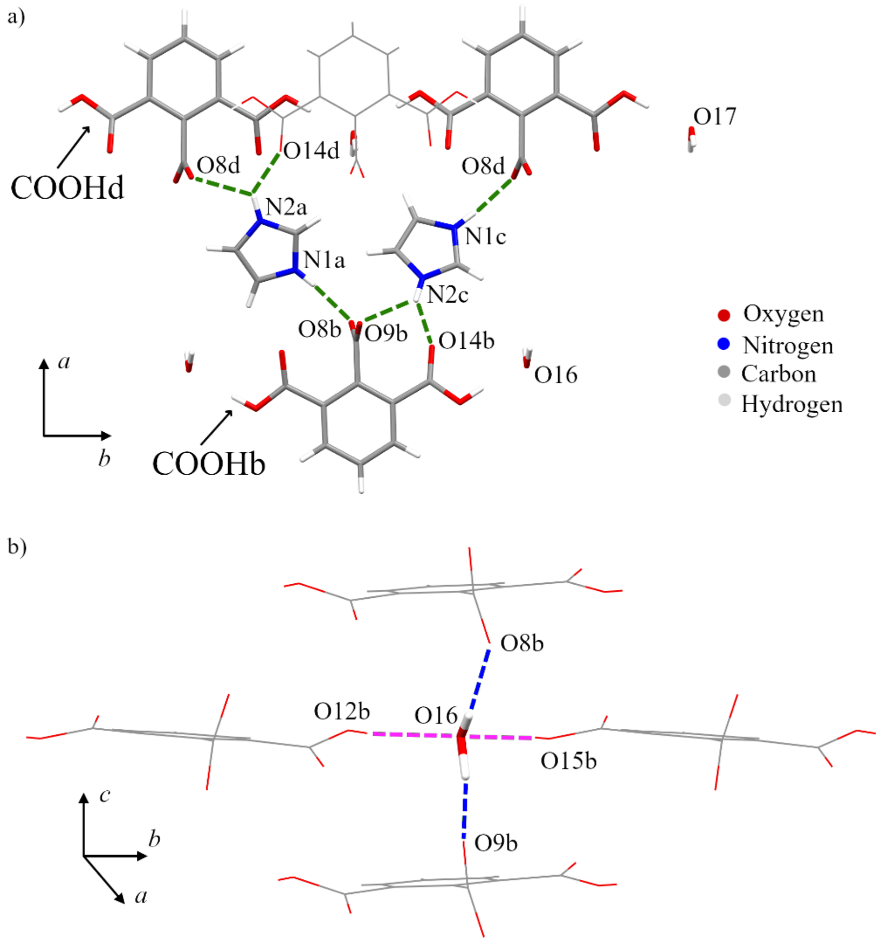

Figure S1. Crystal structure of HemImi·H_2O with $N^+–H\cdots O^-$ hydrogen bonds (green dashed lines) connecting cations and anions (a) and $O–H\cdots O^-$ (blue dashed lines) and $O–H\cdots O$ (pink dashed lines) connecting anions with water molecules (b). It is based on the crystallographic structure CCDC: 2287974.[8]

Table S1. THz, FTIR, and Raman bands at room temperature with the assignment based on the literature.

	FTIR	Raman	Assignment	Literature
1	3309m		νO–H (H_2O)	8–10
2	3190w	3192m	νO–H (anion)	11
3	3170s	3171w	νO–H (anion)	11
4		3159vw	νN–H (cation)	12,13
5	3108m	3112s	νN–H (cation)	12,13
6		3045w	νC–H	12
7	3012w	3017w	νC–H	12
8	1737vs	1743vs	νC=O	8
9	1715s	1713vw	νC=O	8
10	1580vs	1590s	νC=C	12
11	1553vs		νCOO_{asym}	14
12	1455w	1462s	νC–N+δN–H	14
13	1378m		νCOO_{sym}	14
14	1301w		νC–O	14
15	1237vvs	1260vvs	νC–O	12
16	1071vw	1074vvs	νC–C	14
17	951w		γO–H···O	8,10
18	916		δCNC	13,15
19	893m	899vvw	γCN–H	13,15
20	872m		δCOO+δCCC	12
21	831w	839m	γC–CH+γCOOH	12
22	771m	765m	γNC–H (cation)	12
23	760m		δCC–H (anion)	12
24	682vvw	682m	δC–H (cation)	12
25	663m		γC–C (anion)	13,15
26	637w	633vvw	δCNC	16
27	312w	313w	ν_9 νOHO + νNHO	13,15
28	291w		ν_8 νOHO^- + νNHO	9,10
29	246vvw		ν_7 νOHO^- (water)	9,10
30	192w	205vw	ν_6 νNHO	13,15
31		178m	$\nu_5$$\delta OHO^-$ + δNHO	9,10
32		102m	ν_4γNHO + γOHO	
33	59m (1.79 THz)		ν_3γNHO + γOHO	13,15
34	47w (1.42 THz)	52w	ν_2 γOHO + δNHO	17
35	34w (1.03 THz		ν_1 γOHO^- + γNHO	10

Legend: ν – stretching vibration, δ and γ - deformation in-plane and out-of-plane vibrations respectively; Vvw – very very weak, vw – very weak, w – weak, m – medium, s – strong, vs – very strong, vvs – very very strong.

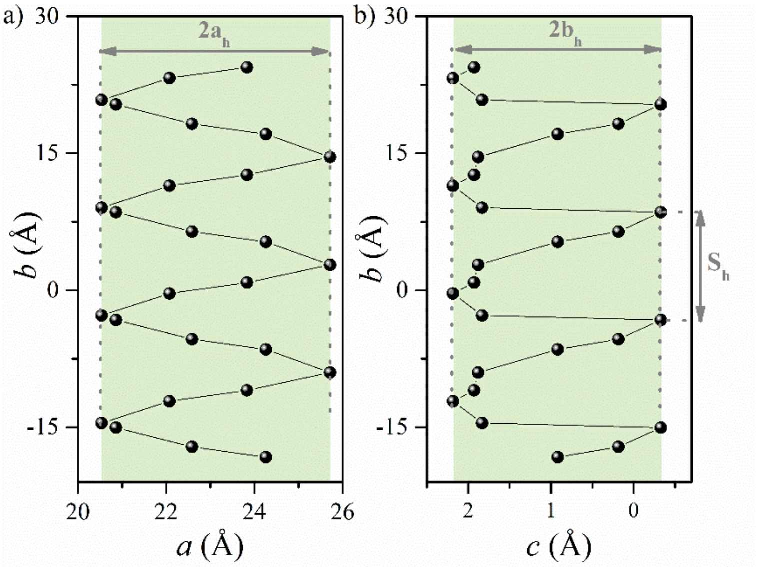

Figure S2. Projection of the helix in the crystal structure of HemImi·H_2O onto the *ab* (a) and *cb* (b) planes. Helix parameters: a_h - semi-major axis, b_h - semi-minor axis, S_h- helix-pitch. The points represent the positions of the donor (nitrogen) and acceptor (oxygen) atoms in N^+–H···O^- hydrogen bonds.

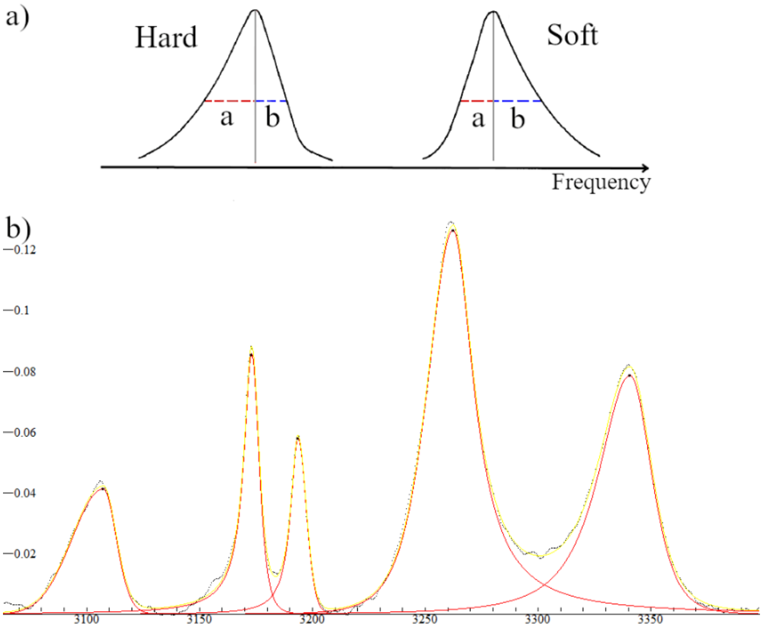

Figure S3. Anharmonic hard and soft force characteristics in the energy absorption curve (a).[18] FT-IR spectrum at 210 K fitted with SplitPearson7 anharmonic functions in the Fityk program in the range of 3000-3400 cm^{-1}.

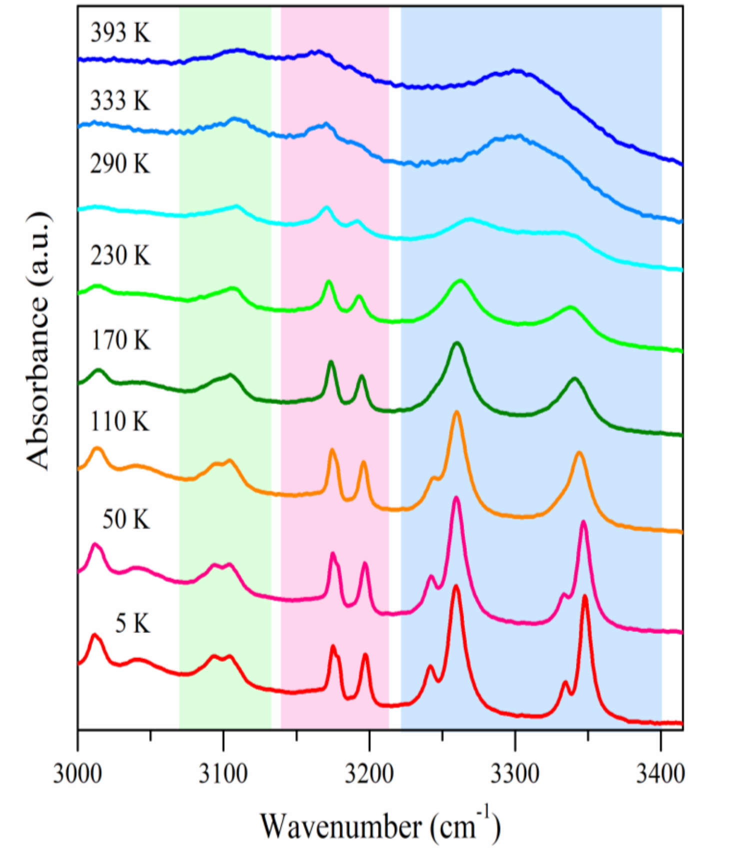

Figure S4. Temperature dependence of FTIR spectra above 3000 cm^{-1} of HemImi·H_2O. Spectra were measured as KBr matrix (*c*=1:500).

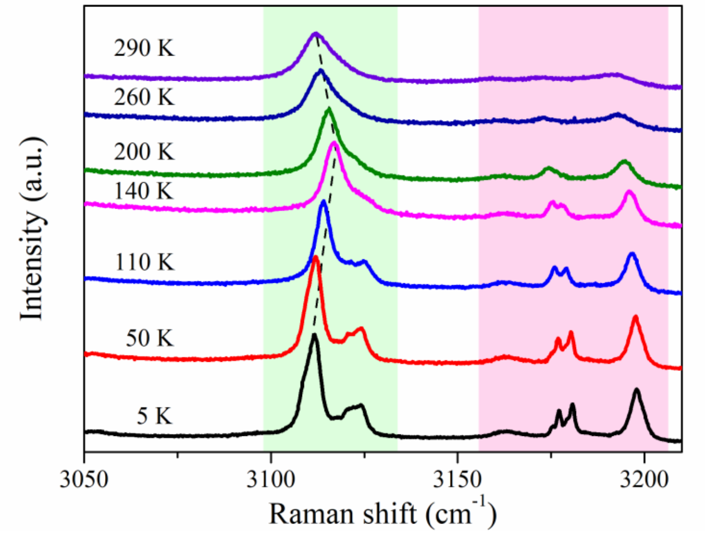

Figure S5. Temperature dependence of Raman spectra above 3000 cm^{-1} of HemImi·H_2O.

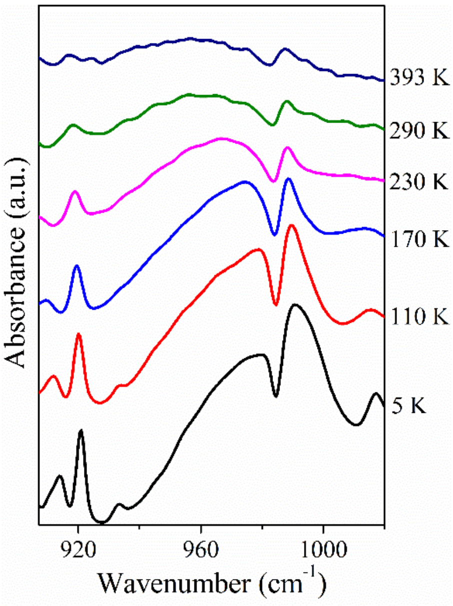

Figure S6. Temperature evolution of experimental FTIR spectra in the range of 910–1020 cm^{-1}.

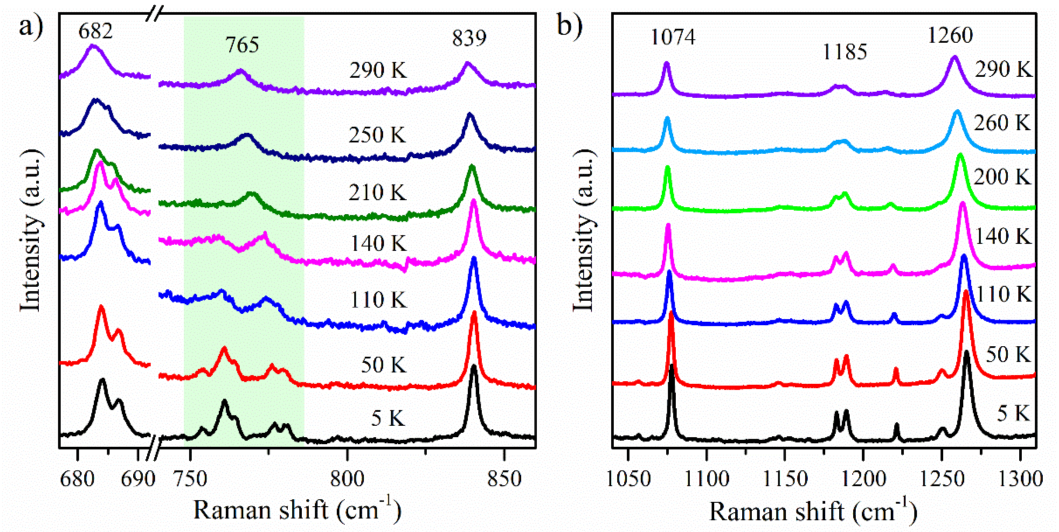

Figure S7. Temperature dependence of Raman spectra in the ranges of 677-860 cm^{-1} (a) and 1040-1310 cm^{-1} (b).

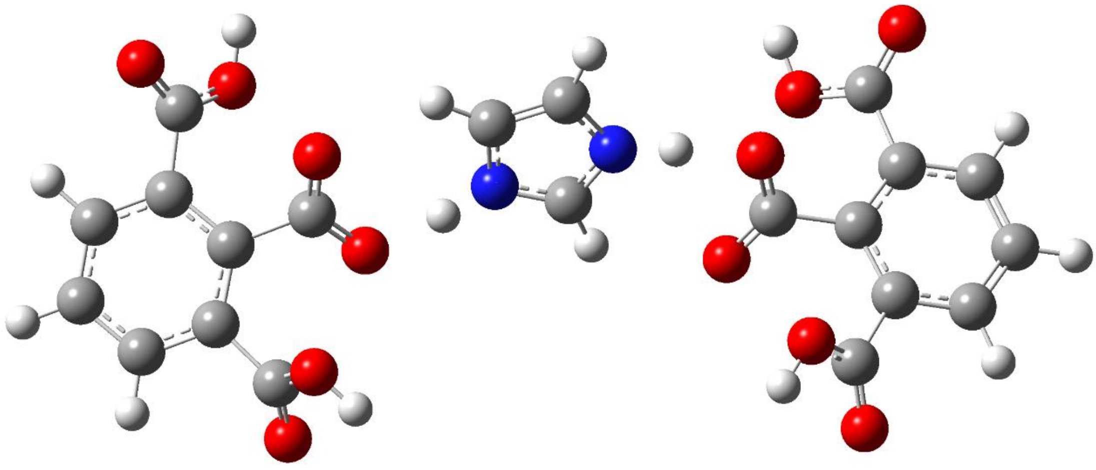

Figure S8. Model system of anions and cation of HemImi·H_2O for which IR and Raman spectra were calculated. Method: DFT/ωB97x-D/6-311G(d,p).

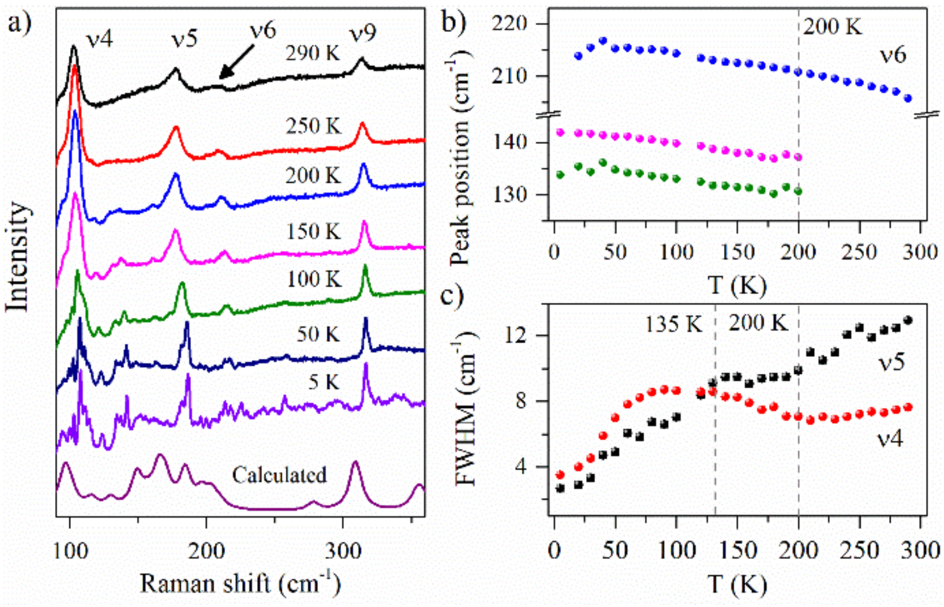

Figure S9. Temperature dependence of experimental and calculated Raman spectra in the range of 90–370 cm^{-1} (a); peak positions in the range of 125-223 cm^{-1} (b), and FWHM of ν4 and ν5 (c).

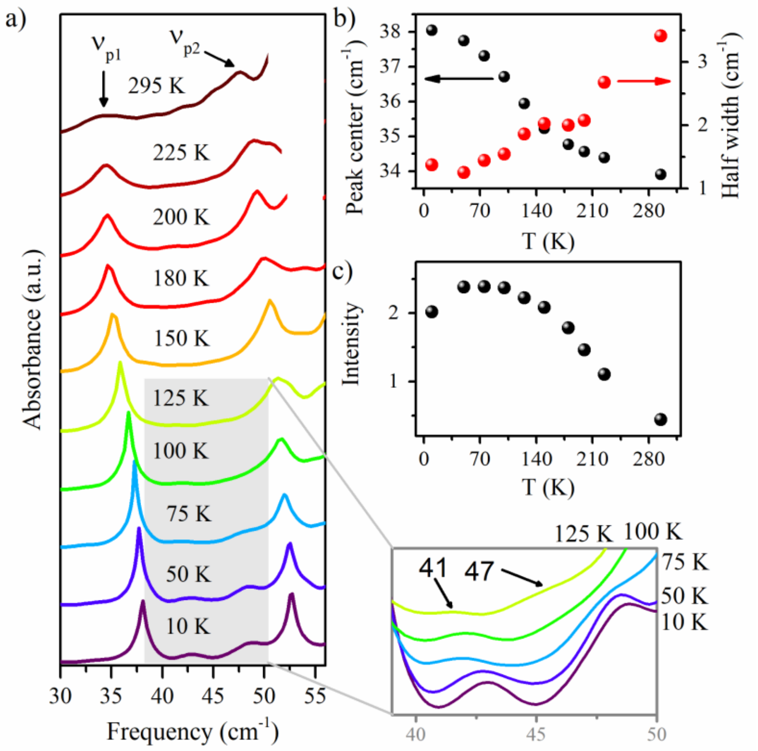

Figure S10. Temperature dependence of THz spectra (a), peak position and FWHM (b) and intensity (c), of ν_1. Measurement was done for the powder sample.

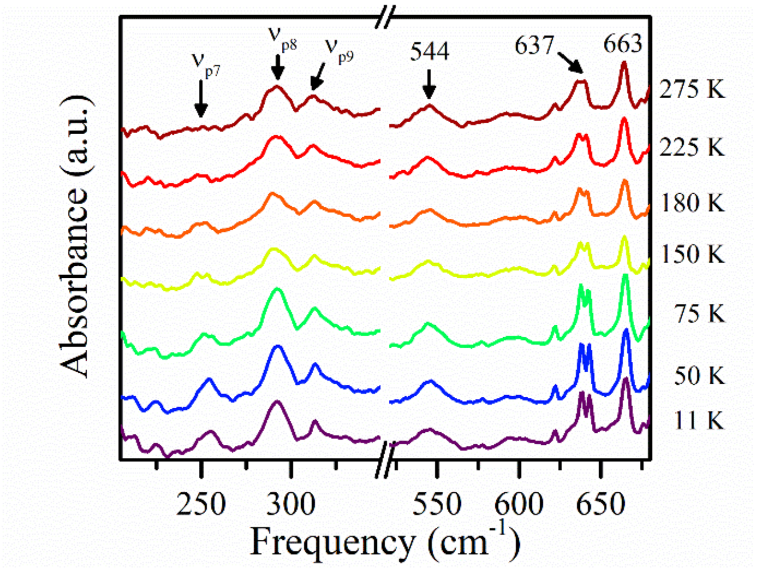

Figure S11. Temperature dependence of FIR spectra. Spectra were measured in Nujol mull.

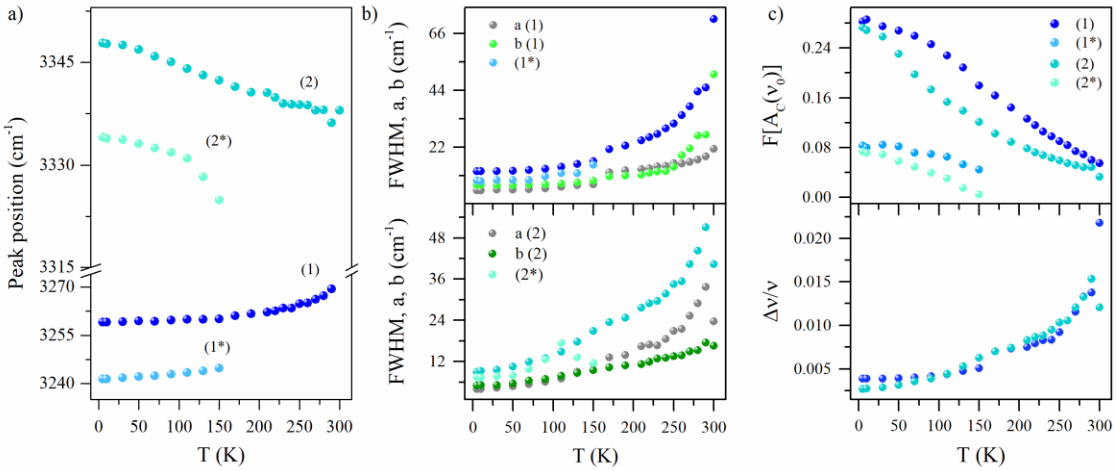

Figure S12. Temperature dependence of peak position (a), FWHM, a, b (see Fig. S5a, b), and maximum energy coefficient $F[A_c(\nu_0)]$ and damping parameters $\Delta\nu/\nu_0$ (c) connected to O–H···O⁻ HBs. These parameters were calculated from an anisotropic shape of bands fitted by SplitPearson7 function in Fityk program.[19]

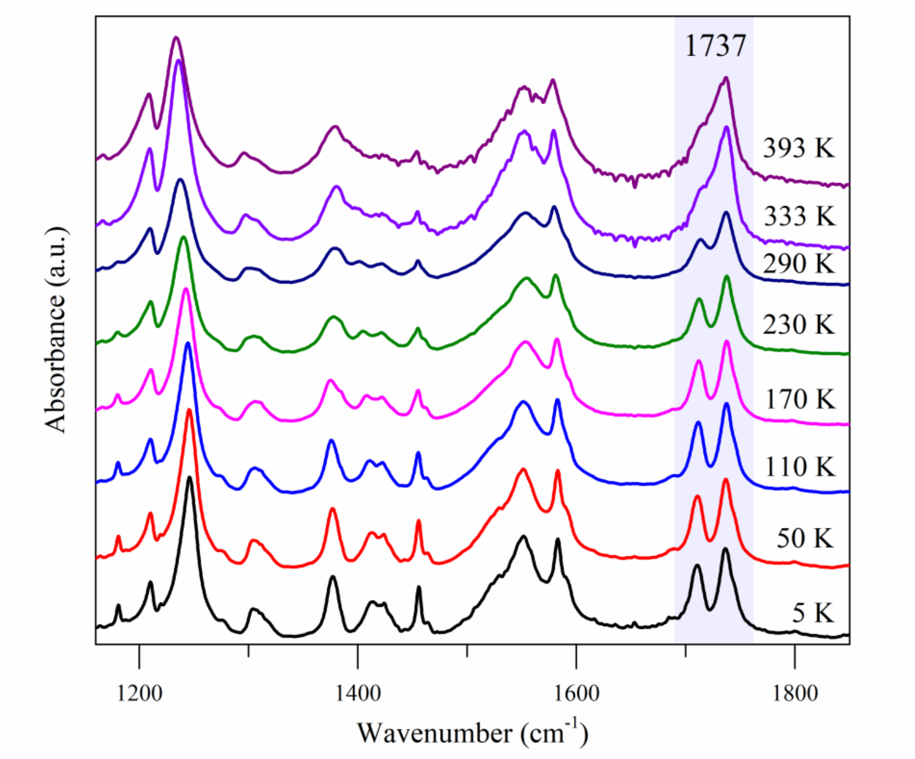

Figure S13. Temperature dependence of MIR spectra in the range of 1160-1850 cm^{-1}.

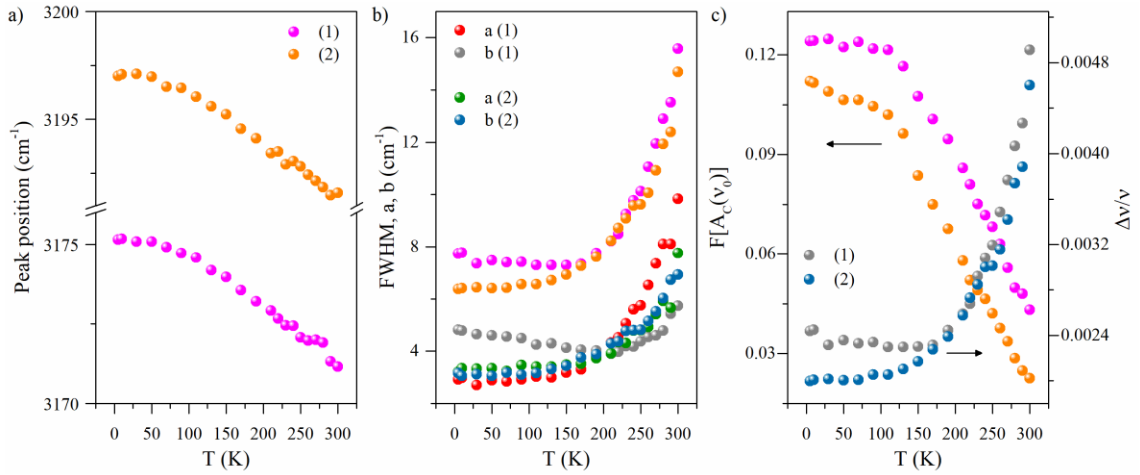

Figure S14. Temperature dependence of peak position (a), FWHM, a, b (see Fig. S5a, b), and maximum energy coefficient $F[A_c(\nu_0)]$ and damping parameters $\Delta\nu/\nu_0$ (c) connected to O–H···O HBs. These parameters were calculated from an anisotropic shape of bands fitted by SplitPearson7 function in Fityk program.[19]

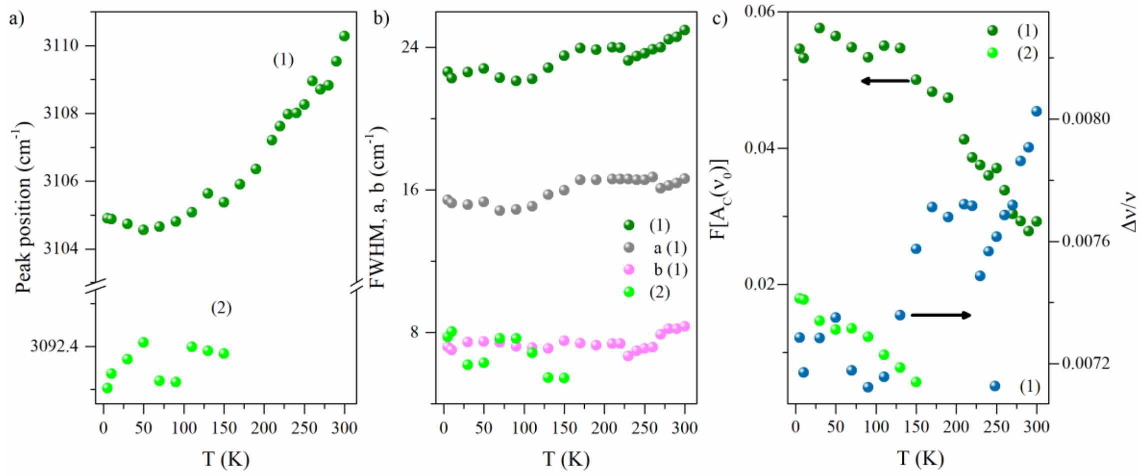

Figure S15. Temperature dependence of peak position (a), FWHM, a, b (see Fig. S5a, b), and maximum energy coefficient F[$A_c(\nu_0)$] and damping parameters $\Delta\nu/\nu_0$ (c) connected to N^+–H···O^- HBs. These parameters were calculated from the anisotropic shape of bands fitted by SplitPearson7 function in Fityk program.[19]

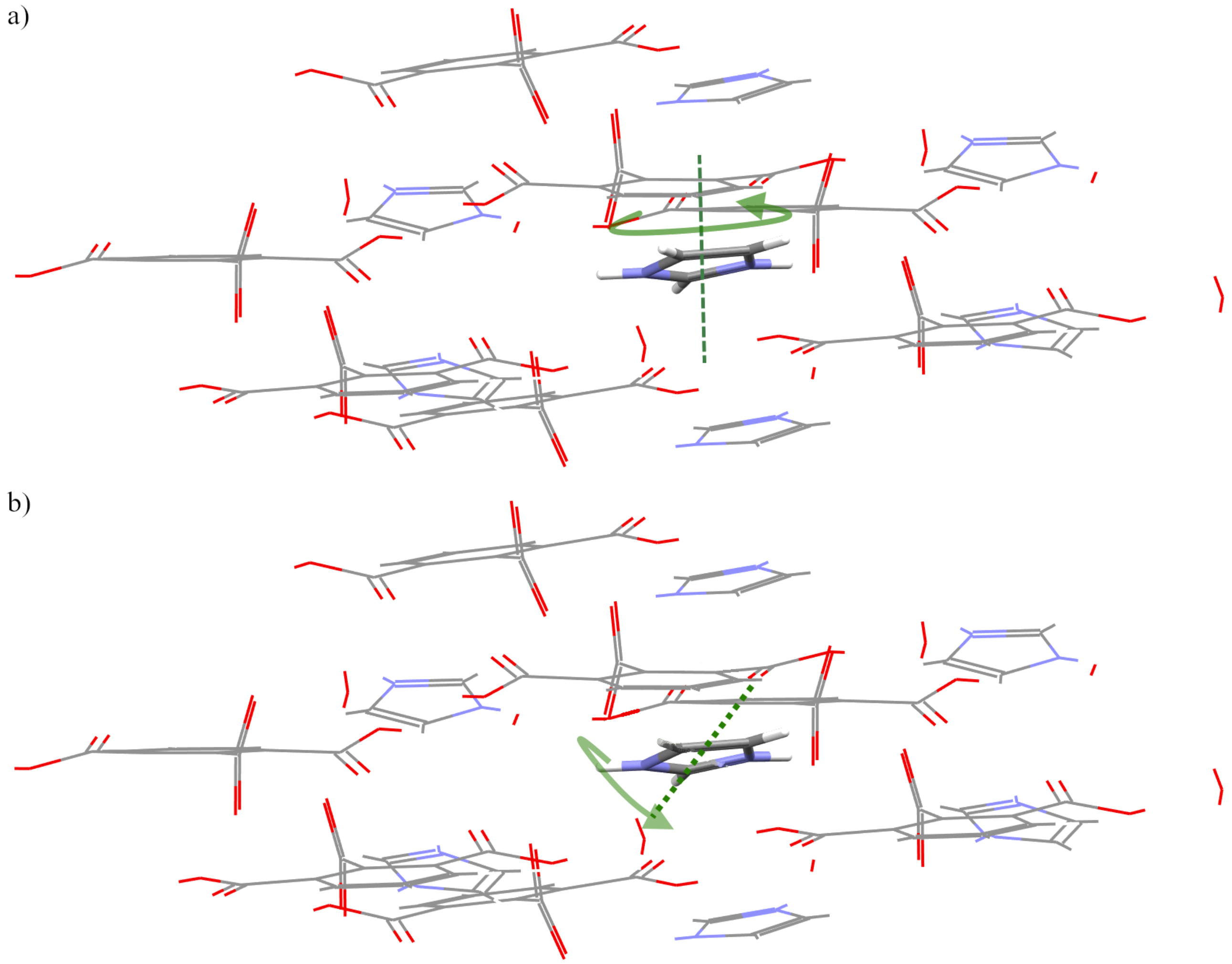

Figure S16. Cation libration (a) and rotation (b) together with the first coordination zones, with the axes of the movement.

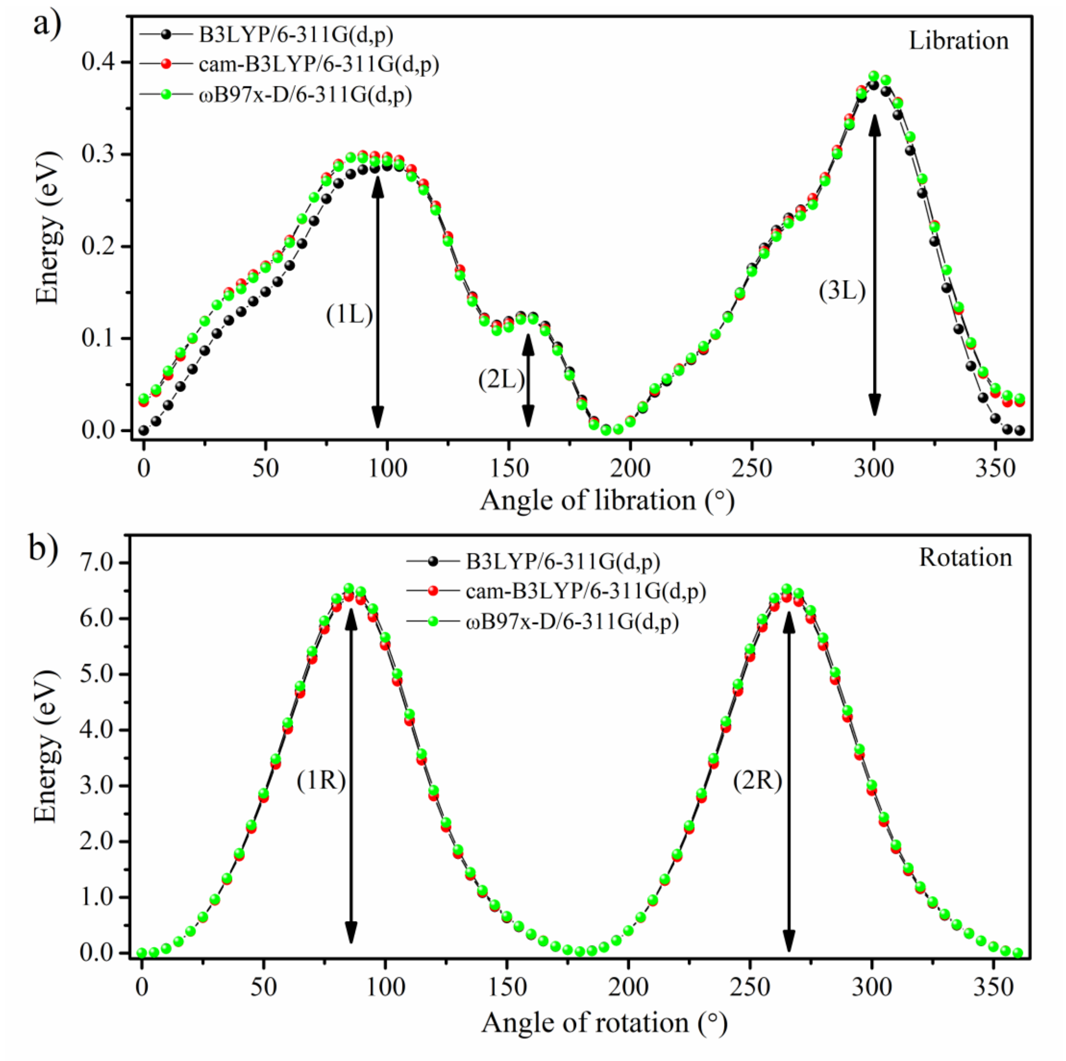

Figure S17. Potential energy scan for an imidazole ion in the case of libration (a) and rotation (b). The calculations were performed using a model system in which the first coordination sphere for imidazole was selected. The calculations were performed using the B3LYP, cam-B3LYP and ωB97x-D functionals together with the Pople Basis Set 6-311G(d,p).

Table S2. Potential barrier values and rotation angles obtained using the PES method for libration (1L, 2L, and 3L) and rotation (1R and 2R) for the model system (see Fig. S16).

	B3LYP/6-311G(d,p)		cam-B3LYP/6-311G(d,p)		ωB97x-D/6-311G(d,p)	
ST	Angle (°)	Energy (eV)	Angle (°)	Energy (eV)	Angle (°)	Energy (eV)
(1L)	100	0.287	90	0.299	85	0.296
(2L)	160	0.123	155	0.122	160	0.121
(3L)	300	0.375	300	0.385	300	0.385
(1R)	85	6.440	85	6.394	85	6.547
(1R)	265	6.428	265	6.381	265	6.535

ST – state transition marked by arrows in Fig. S15.

Reference

(1) Frisch, M. J.; Trucks, G. W.; Schlegel, H. B.; Scuseria, G. E.; Robb, M. A.; Cheeseman, J. R.; Scalmani, G.; Barone, V.; Petersson, G. A.; Nakatsuji, H.; Li, X.; Caricato, M.; Marenich, A.; Bloino, J.; Janesko, B. G.; Gomperts, R.; Mennucci, B.; Hratchian, H. P.; Ortiz, J. V.; Izmaylov, A. F.; Sonnenberg, J. L.; Williams-Young, D.; Ding, F.; Lipparini, F.; Egidi, F.; Goings, J.; Peng, P.; Petrone, A.; Henderson, T.; Ranasinghe, D.; Zakrzewski, V. G.; Gao, J.; Rega, N.; Zheng, G.; Liang, W.; Hada, M.; Ehara, M.; Toyota, K.; Fukuda, R.; Hasegawa, J.; Ishida, M.; Nakajima, T.; Honda, Y.; Kitao, O.; Nakai, H.; Vreven, T.; Throssell, K.; Montgomery, J. A.; Peralta, Jr. J. E.; Ogliaro, F.; Bearpark, M.; Heyd, J. J.; Brothers, E.; Kudin, K. N.; Staroverov, V. N.; Keith, T.; Kobayashi, R.; Normand, J.; Raghavachari, K.; Rendell, A.; Burant, J. C.; Iyengar, S. S.; Tomasi, J.; Cossi, M.; Millam, J. M.; Klene, M.; Adamo, C.; Cammi, R.; Ochterski, J. W.; Martin, R. L.; Morokuma, K.; Farkas, O.; Foresman, J. B.; Fox, D. J. Gaussian, Inc., Wallingford CT, 2016.

(2) Kim, K.; Jordan, K. D. Comparison of Density Functional and MP2 Calculations on the Water Monomer and Dimer. *J. Phys. Chem.* **1994**, *98* (40), 10089–10094. https://doi.org/10.1021/j100091a024.

(3) Stephens, P. J.; Devlin, F. J.; Chabalowski, C. F.; Frisch, M. J. Ab Initio Calculation of Vibrational Absorption and Circular Dichroism Spectra Using Density Functional Force Fields. *J. Phys. Chem.* **1994**, *98* (45), 11623–11627. https://doi.org/10.1021/j100096a001.

(4) Becke, A. D. Density-Functional Thermochemistry. III. The Role of Exact Exchange. *J. Chem. Phys.* **1993**, *98* (7), 5648–5652. https://doi.org/10.1063/1.464913.

(5) Yanai, T.; Tew, D. P.; Handy, N. C. A New Hybrid Exchange–Correlation Functional Using the Coulomb-Attenuating Method (CAM-B3LYP). *Chemical Physics Letters* **2004**, *393* (1–3), 51–57. https://doi.org/10.1016/j.cplett.2004.06.011.

(6) Chai, J.-D.; Head-Gordon, M. Systematic Optimization of Long-Range Corrected Hybrid Density Functionals. *J. Chem. Phys.* **2008**, *128* (8). https://doi.org/10.1063/1.2834918.

(7) Ditchfield, R.; Hehre, W. J.; Pople, J. A. Self-Consistent Molecular-Orbital Methods. IX. An Extended Gaussian-Type Basis for Molecular-Orbital Studies of Organic Molecules. *J. Chem. phys.* **1971**, *54* (2), 724–728. https://doi.org/10.1063/1.1674902.

(8) Zięba, S.; Mizera, A.; Markiewicz, K. H.; Dubis, A. T.; Ławniczak, P.; Gzella, A.; Siergiejczyk, L.; Łapiński, A. Effect of Azole Counterions on Thermal and Transport Properties of the Hydrated Salts of Hemimelitic Acid. *J. Phys. Chem. C* **2023**, *127* (50), 24403–24410. https://doi.org/10.1021/acs.jpcc.3c05815.

(9) Hetmańczyk, J.; Hetmańczyk, Ł.; Migdał-Mikuli, A.; Mikuli, E.; Wesełucha-Birczyńska, A. Raman Light Scattering, Infrared Absorption and DSC Studies of the Phase Transition and Vibrational and Reorientational Dynamics of H_2 O Ligands and ClO_4^- Anions in $[Ba(H_2O)_3](ClO_4)_2$. *J Raman Spectroscopy* **2012**, *43* (8), 1118–1125. https://doi.org/10.1002/jrs.3130.

(10) Flór, M.; Wilkins, D. M.; De La Puente, M.; Laage, D.; Cassone, G.; Hassanali, A.; Roke, S. Dissecting the Hydrogen Bond Network of Water: Charge Transfer and Nuclear Quantum Effects. *Science* **2024**, *386* (6726), eads4369. https://doi.org/10.1126/science.ads4369.

(11) Ishii, K.; Takeuchi, S.; Tahara, T. Infrared-Induced Coherent Vibration of a Hydrogen-Bonded System: Effects of Mechanical and Electrical Anharmonic Couplings. *J. Chem. Phys.* **2009**, *131* (4), 044512. https://doi.org/10.1063/1.3181777.

(12) Deng, Z.; Wang, Y.; Qi, G.; Zhang, Q. High-Pressure Structural Stability and Melting Performance of α-2,4-Dinitroanisole. *Energetic Materials Frontiers* **2021**, *2* (4), 272–277. https://doi.org/10.1016/j.enmf.2021.11.001.

(13) Zięba, S.; Dubis, A. T.; Rusek, M.; Katrusiak, A.; Gzella, A.; Łapiński, A. Negative Thermal Expansion and Linear Compressibility in 1 *H* -Imidazol-3-Ium 2-Hydroxybenzoate with a Helical Network of Hydrogen Bonds. *Phys. Chem. Chem. Phys.* **2025**, *27* (16), 8559–8569. https://doi.org/10.1039/D4CP04519J.

(14) Bajaj, N.; Bhatt, H.; Vishwakarma, S. R.; Deo, M. N. Orientational Adaptations Leading to Plausible Phase Transitions in L -Leucine at Low Temperatures: Revealed by Infrared Spectroscopy. *J. Phys. Chem. B* **2019**, *123* (3), 561–570. https://doi.org/10.1021/acs.jpcb.8b09590.

(15) Zięba, S.; Gzella, A.; Dubis, A. T.; Łapiński, A. Combination of Negative, Positive, and Near-Zero Thermal Expansion in Bis(Imidazolium) Terephthalate with a Helical Hydrogen-Bonded Network. *Cryst. Growth Des.* **2021**, *21* (7), 3838–3849. https://doi.org/10.1021/acs.cgd.1c00167.

(16) Zięba, S.; Mizera, A.; Pogorzelec-Glaser, K.; Łapiński, A. Proton Conducting System (ImH 2) 2 SeO 4 ·2H 2 O Investigated with Vibrational Spectroscopy. *Spectrochimica Acta Part A: Molecular and Biomolecular Spectroscopy* **2017**, *180*, 224–233. https://doi.org/10.1016/j.saa.2017.02.049.

(17) Matsui, H.; Shimatani, K.; Ikemoto, Y.; Sasaki, T.; Matsuo, Y. Phonon-Assisted Proton Tunneling in the Hydrogen-Bonded Dimeric Selenates of Cs3H(SeO4)2. *J. Chem. Phys.* **2020**, *152* (15), 154502. https://doi.org/10.1063/1.5145108.
(18) Plendl, J. N. Some New Interrelations in the Properties of Solids Based on Anharmonic Cohesive Forces. *Phys. Rev.* **1961**, *123* (4), 1172–1180. https://doi.org/10.1103/PhysRev.123.1172.
(19) Wojdyr, M. *Fityk* : A General-Purpose Peak Fitting Program. *J Appl Crystallogr* **2010**, *43* (5), 1126–1128. https://doi.org/10.1107/S0021889810030499.
